# Conformational Changes in Protein Loops and Helices Induced by Post-Translational Phosphorylation

**DOI:** 10.1371/journal.pcbi.0020032

**Published:** 2006-04-21

**Authors:** Eli S Groban, Arjun Narayanan, Matthew P Jacobson

**Affiliations:** 1 Department of Pharmaceutical Chemistry, University of California San Francisco, San Francisco, California, United States of America; 2 Graduate Group in Biophysics, University of California San Francisco, San Francisco, California, United States of America; Harvard University, United States of America

## Abstract

Post-translational phosphorylation is a ubiquitous mechanism for modulating protein activity and protein-protein interactions. In this work, we examine how phosphorylation can modulate the conformation of a protein by changing the energy landscape. We present a molecular mechanics method in which we phosphorylate proteins in silico and then predict how the conformation of the protein will change in response to phosphorylation. We apply this method to a test set comprised of proteins with both phosphorylated and non-phosphorylated crystal structures, and demonstrate that it is possible to predict localized phosphorylation-induced conformational changes, or the absence of conformational changes, with near-atomic accuracy in most cases. Examples of proteins used for testing our methods include kinases and prokaryotic response regulators. Through a detailed case study of cyclin-dependent kinase 2, we also illustrate how the computational methods can be used to provide new understanding of how phosphorylation drives conformational change, why substituting Glu or Asp for a phosphorylated amino acid does not always mimic the effects of phosphorylation, and how a phosphatase can “capture” a phosphorylated amino acid. This work illustrates how computational methods can be used to elucidate principles and mechanisms of post-translational phosphorylation, which can ultimately help to bridge the gap between the number of known sites of phosphorylation and the number of structures of phosphorylated proteins.

## Synopsis

Many proteins are chemically modified after they are synthesized in the cell. These post-translational modifications can modulate the ability of a protein to perform chemical reactions and to interact with other proteins. At the cellular level, for example, these chemical modifications are critical for allowing the cell to respond to its environment and control its division. One of the most common mechanisms by which proteins can be modified is by phosphorylation—the addition of a phosphate group to an amino acid side chain of the protein. Thousands of proteins are known to be modified by phosphorylation, but only for a small minority of these do we have any detailed understanding of how the chemical modification regulates the function of the protein. The authors describe a computational method that can make testable predictions about the structural changes that occur in a protein induced by post-translational phosphorylation. Their results show that the method can produce structural models of the phosphorylated proteins with near-atomic accuracy, and provide insight into the energetics of conformational switches driven by phosphorylation. As such, the computational method complements experiments aimed at understanding the mechanisms of protein regulation by phosphorylation.

## Introduction

Post-translational phosphorylation is a ubiquitous mechanism for cellular regulation, playing a role in such diverse processes as signal transduction, transport, cytoskeletal regulation, and metabolism. A variety of amino acids can be phosphorylated, but serine, threonine, and tyrosine are the most important sites of phosphorylation in eukaryotes, whereas histidine and aspartate play the central role in the “two-component” signaling pathways of prokaryotes. Several thousand sites of post-translational phosphorylation are now known [1], and this number will continue to grow quickly. Estimates of the fraction of proteins that are phosphorylated in vivo range as high as 30% [1]; higher values are associated with particular stages of the cell cycle or responses to external stimuli. Protein kinases catalyze post-translational phosphorylation, and many kinases are themselves regulated by phosphorylation, leading to complex signaling and regulatory networks. Kinases are targets of aggressive drug development efforts [2] aimed at treating cancer and other diseases such as diabetes.

Despite the huge amount of research related to post-translational phosphorylation, the detailed role that specific sites of post-translational phosphorylation play in the function of individual proteins remains poorly understood in most cases. Structural information is particularly limited, due in part to the difficulty of obtaining sufficient purified protein in a specific modification state. X-ray crystallography has determined atomic-resolution structures for a few tens of phosphorylated proteins [3], whereas nuclear magnetic resonance (NMR) experiments have elucidated structure and dynamics of a similar number of phosphorylated peptides and small proteins in solution [4–10]. Electron paramagnetic resonance (EPR) and circular dichroism experiments can also provide information on conformational change due to phosphorylation [11], but do not provide atomic detail. The number of known phosphorylation sites will almost certainly continue to grow much more quickly than the number of structurally well-characterized phospho-proteins.

We believe that computational studies can play a central role in elucidating principles and mechanisms of post-translational modification, and ultimately help to bridge the gap between the number of known sites of phosphorylation and the number of structures of phosphorylated proteins. Only a small number of computational modeling studies have been published on protein phosphorylation, particularly in relation to the thousands of molecular/cellular biology studies published every year on the topic. A few studies have focused on model systems, including studies from the McCammon group that examined the role of phosphorylation in stabilizing the N-termini of helices [12] and conformational/dynamical changes due to phosphorylation of a Ser residue in a tetrapeptide [13,14]. Luo et al. investigated the strengths of solvent-exposed salt bridges, including model systems for phosphate interactions with lysine and arginine, using a Generalized Born solvent model [15]. A few modeling studies of specific phosphorylated proteins have also been reported. Maurer et al. investigated the influence of phosphorylation on the docking of a peptide to bovine thrombin [16]. Zhou and Abagyan calculated the binding energy of phosphotyrosine-containing peptides to SH2 and PTB domains [17]. Several molecular dynamics [18–30] and homology modeling [27,31,32] studies have been reported involving phosphorylated proteins [18–20,24,25,31–33] or peptides [21–23,26,27,34]. A Car-Parinello study has been reported [35], as well as a creative application of docking algorithms to investigate conformational changes upon phosphorylation of the N-terminal tail in phenylalanine hydroxylase [36].

**Figure 1:**
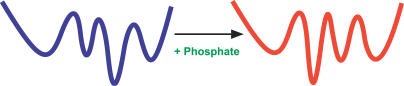
Phosphorylation Can Perturb the Energy Landscape of a Protein to Cause Changes in Conformation and Dynamics This figure is a visual representation of such changes. In this work, we predict the structural change of proteins due to phosphorylation by locating the new global energy minimum of the energy surface.

In this work, we examine how phosphorylation can modulate the conformation of a protein by changing the energy landscape. We limit our attention in this work to relatively small conformational changes involving a phosphorylated loop and its surroundings, and in one case, conformational changes in a helix and its surroundings. At physiological pH, the phosphate group predominantly carries a −2 charge, whereas the site of phosphorylation is neutral before modification (in the case of Ser, Thr, and Tyr). The conceptual foundation of our work is shown in Figure 1. We view the phosphate as a perturbation to the energy landscape of the protein. Our goal in this work is to predict the conformational changes caused by this perturbation. That is, given the structure (or an accurate model) of the unphosphorylated protein, can we predict the structure of the phosphorylated protein? The critical tools we need to understand and predict the structural effects of post-translational phosphorylation are as follows:

One tool that is needed is an energy function that captures the essential physics, especially for the modified residues. We use a molecular mechanics energy function consisting of the OPLS-AA force field and a Generalized Born implicit solvent model. The use of a physics-based energy function is important for two reasons. First, there are too few structures of phosphorylated proteins to make knowledge-based energy functions feasible. Second, conformational changes induced by phosphorylation are largely driven by the electrostatic perturbation induced by the phosphate group. We use the energy function not only to predict conformational changes induced phosphorylation, but also to better understand the key physical effects underlying these conformational changes, especially in our case study of cyclin dependent kinase 2 (CDK2).

Another necessary tool is sampling algorithms capable of exploring critical degrees of freedom. After phosphorylating a protein in silico, we need to explore the new energy landscape and identify the new global energy minimum, if in fact it is significantly different than that of the unphosphorylated protein. In principle, molecular dynamics could be used for this purpose, and in some cases, this type of strategy has been successful, in studies by ourselves and others [18–30]. However, the timescales for converting from the non-phospho to the phosphorylated form are unknown, and in fact are not physically meaningful because the in silico phosphorylation is alchemical, i.e., we do not attempt to mimic a kinase actually performing the phosphorylation. For relatively large conformational changes, or those involving high-energy barriers, e.g., Pro peptide bond isomerization, the relevant timescale could be quite long—microseconds or more. Instead, we use a strategy in which we have adapted algorithms that we previously developed for homology modeling, i.e., sampling methods for side chains [37,38], loops [39–41], and helices [42]. The essence of the approach is to combine dihedral angle sampling methods, which enable large energy barriers to be surmounted, with direct minimization to enumerate many local minima. We discuss our strategy in detail in Materials and Methods.

The key conclusion of this study is that our molecular mechanics–based methods appear to be capable of reproducing conformational changes induced by post-translational phosphorylation, with near-atomic resolution in most cases considered here, which are limited to relatively modest conformational changes and not, e.g., more drastic order–disorder transitions. This work thus represents a significant step toward a broadly applicable method for predicting structural effects of phosphorylation. Through a detailed case study of CDK2, we also illustrate how the computational methods can be used to provide new understanding of how phosphorylation drives conformational change, why substituting Glu or Asp for a phosphorylated amino acid does not always mimic the effects of phosphorylation, and how a phosphatase can “capture” a phosphorylated amino acid.

## Results/Discussion

We first present an in-depth study of activation loop phosphorylation in CDK2, both to introduce the computational methods and to highlight the ability of these methods to gain new insights into how phosphorylation drives conformational changes. We then present a broader survey of our ability to predict phosphorylation-driven conformational changes.

### Case Study: CDK2

CDK2 is a member of the cyclin-dependent kinase protein family [43], whose members play a central role in cell cycle regulation. CDK2 is activated through binding of cyclin A and post-translational phosphorylation of a threonine residue on the activation loop, which lies close to the catalytic site. Compared to other phosphorylated proteins, there is a wealth of structural information for CDK2 in both its phosphorylated and unphosphorylated forms [3]. In this case study, we examine the ability of our method to: (1) reconstruct and predict the active conformation of the activation loop in CDK2 bound to cyclin A [44]; (2) reconstruct the cyclin A dependence of CDK2 activation [44,45]; (3) discriminate between two potential phosphate localization sites in the CDK2/kinase-associated phosphatase (KAP) complex [46]; and (4) examine the effects of substituting Thr160 with a Glu, which results in only partial activation relative to phosphorylation of Thr160 [47].

A structural alignment and superposition of the active and inactive [48] forms of CDK2 bound to cyclin A reveals that the global Cα root mean square deviation (RMSD) between the two structures is only 0.5 Å if residues 152–163 are removed from the calculation. The phosphorylatable threonine residue itself moves approximately 10 Å upon phosphorylation, as measured at the γ oxygen. In the unphosphorylated structure, Thr160 is well solvated and somewhat disordered (B-factors > 50). In the phosphorylated structure, pThr160 localizes to interact with a cluster of Arg residues (50, 126, and 150). We will refer to this and similar conformations as “active” conformations of the loop.

**Figure 2:**
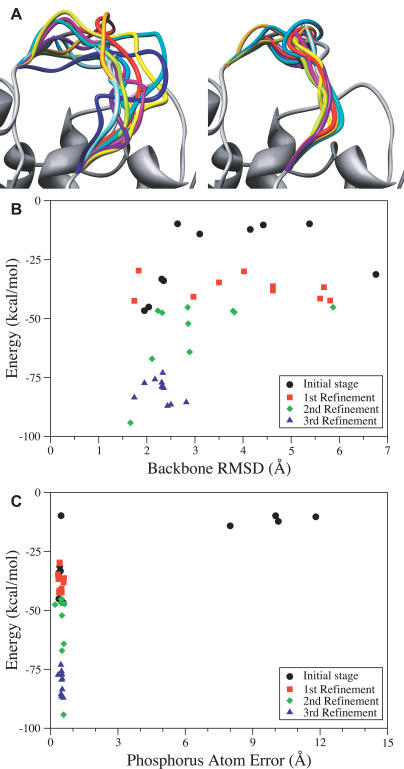
Example of the Hierarchical Loop Prediction, Applied to Reconstructing the Phosphorylated Activation Loop of CDK2/Cyclin A In this example, only the loop is predicted, and the remainder of the protein is held rigid in its crystallographic conformation. (A) Backbone traces for the ten lowest-energy loops sampled in the initial build-up stage (left) and the third and final refinement stage (right) of the hierarchical loop prediction protocol (figures prepared with Chimera [72]). (B) Energies as a function of backbone RMSD values of the 20 lowest-energy loops sampled in the four stages of the loop prediction protocol. (C) Energies as a function of the error in the phosphorus atom position, relative to the crystal structure, for the 20 lowest-energy loops sampled in the four stages of the loop prediction protocol.

As described in Materials and Methods, we use a hierarchical loop prediction algorithm [40] based upon dihedral angle backbone sampling, rotamer-based side chain optimization, an all-atom force field and a Generalized Born solvation model [49,50] to predict the structural consequences of phosphorylation on loops and their surroundings. The hierarchical prediction algorithm allows us to quickly prune the conformational space and focus sampling on energetically favorable regions of conformation space (Figure 2). Unlike Monte Carlo and molecular dynamics sampling schemes, the algorithm itself has no knowledge of the starting conformation of the loop. The entire conformational space of the loop is sampled with varying sampling resolution in a hierarchical manner. This method has proven successful in recreating crystallographic conformations of unphosphorylated loops to loop lengths of 12 residues. In this paper, we expand on this loop prediction method to predict the structures of phosphorylated loops.

Before attempting to predict the phosphorylated structure from the unphosphorylated structure, we first ensure that we can predict the conformation of the phosphorylated loop with high accuracy when all other portions of the phosphorylated structure are taken from the crystal structure. We refer to this test as “loop reconstruction.” This tests the suitability of the energy function for identifying the correct conformation of the phosphorylated loop and the suitability of the sampling function for generating native-like structures. Much of our analysis will focus on the accuracy with which the phosphate group is positioned, which is measured by the distance of the phosphorus atom to its crystallographic position. We also provide other measures of accuracy for the overall loop, including a backbone RMSD measure (calculated based on the N, Cα, and C atoms), and the RMSD calculated over all heavy atoms, including side chains. For all of these measures of accuracy, the predicted structure is first aligned to the reference crystal structure by a least-squares superposition of all Cα atoms, excluding the loop being simulated. Thus, structural differences outside of the loop region being predicted can affect the reported measures of accuracy.

**Figure 3:**
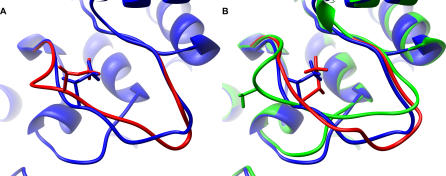
Loop Reconstruction and Prediction in CDK2/Cyclin A (A) Reconstruction: Crystal structure of phosphorylated CDK2/cyclin A (blue) and the predicted loop structure (red). The starting structure for the prediction was the phosphorylated structure, with the only difference being the loop region. (B) Prediction: Crystal structure of phosphorylated CDK2/cyclin A (blue), the crystal structure of the unphosphorylated CDK2/cyclin A (green), and the predicted loop structure upon in silico phosphorylation of the unphosphorylated CDK2/cyclin A structure (red). Figures prepared with Chimera [72].

For the complex of phosphorylated CDK2 with cyclin A, we are able to reproduce the conformation of the activation loop with less than 2 Å RMSD overall. The phosphate group is placed with particularly high accuracy, less than 1 Å error as measured by the position of the phosphorous atom (Figures 2 and 3, and Table 1). We also wish to highlight the differences between Figure 2B and 2C, which plot the energy of different local minima identified during the four-stage prediction algorithm versus the backbone RMSD (Figure 2B) and the error in the phosphate position (Figure 2C). In the energy versus backbone RMSD plot, an energy “funnel” is clearly evident. That is, the lowest energy identified decreases as the loop, as a whole, approaches native-like conformations. On the other hand, the phosphate group itself appears to respond to a narrow, deep energy well. That is, the phosphate locates the Arg cluster very early in the simulation (during the first of the four stages of the loop prediction algorithm), and remains there while the rest of the loop adjusts to the new position of the phosphate group.

**Table 1:**
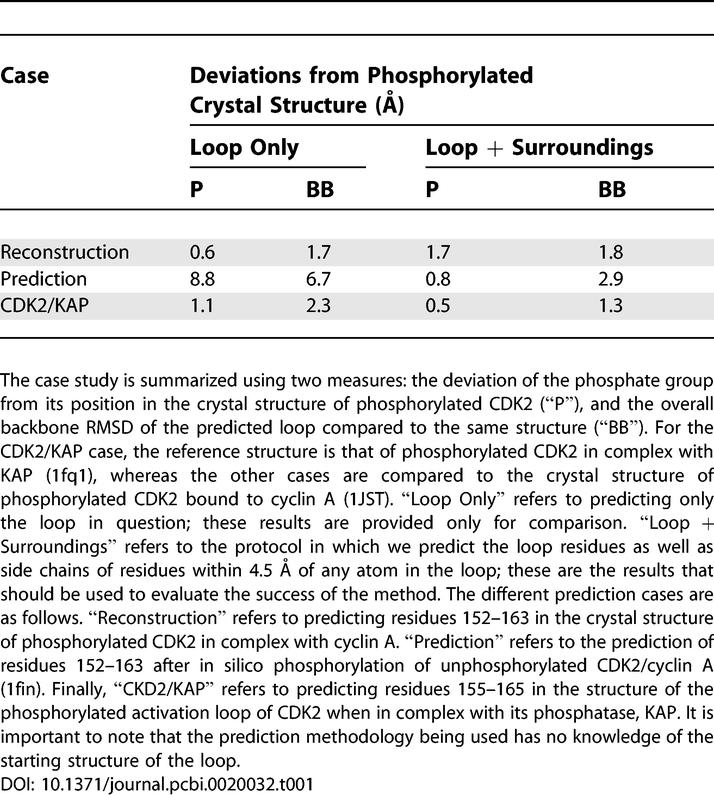
Results from the CDK2 Case Study

**Figure 4:**
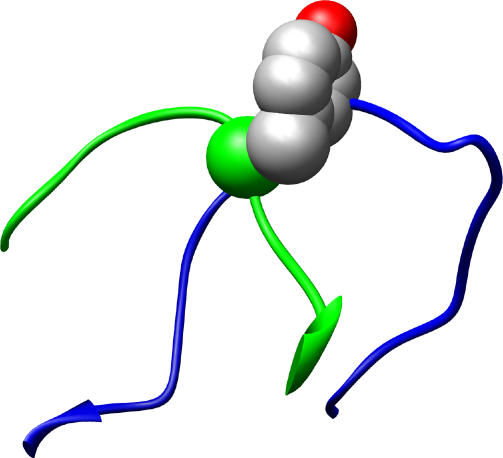
Detailed View of One Portion of the Structural Superposition between the Phosphorylated and Unphosphorylated Crystal Structures of CDK2/Cyclin A The phosphorylated activation loop (blue) passes through the middle of Tyr179 (CPK) when inserted into the non-phosphorylated structure (green) of CDK2. Figure prepared with Chimera [72].

Next, we examine our ability to predict the phosphorylated loop conformation starting from the unphosphorylated structure. Naively attempting to predict the conformation of the phosphorylated activation loop from the unphosphorylated structure, holding the nearby side chains fixed, results in a poor prediction, with an error in the position of the phosphorus atom of approximately 9 Å and a backbone RMSD of approximately 7 Å (Table 1). The reason for this failure is clear. A comparison of the phosphorylated and unphosphorylated structures reveals that the backbone of the phosphorylated conformation of the activation loop passes directly through the side chain of Tyr179 in the unphosphorylated form (Figure 4). This single misplaced side chain can prevent the loop from assuming the correct activated conformation. Thus, in this case, introduction of the phosphate group not only changes the activation loop conformation, but also rearranges the side chain hydrogen bonding network well beyond the loop itself.

**Figure 5:**
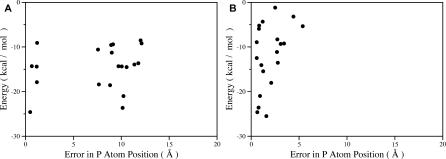
CDK2 Case Study The 20 lowest-energy loops predicted for (A) reconstruction of residues 152–163 in phosphorylated, cyclin-bound CDK2; and (B) prediction of the structure of residues 152–163 upon in silico phosphorylation of Thr160 in the unphosphorylated, cyclin-bound CDK2. In each case, the loop and surrounding side chains are optimized simultaneously as described in Materials and Methods.

Thus, sampling of loop conformations must be coupled with, at least, the sampling of side chain conformations in the vicinity of the loop to permit accurate prediction. As described in Materials and Methods, we have adopted a strategy in which side chains near the loop are removed during the loop build-up, and side chains both on the loop and its vicinity are optimized and energy minimized simultaneously for the representative loop from each cluster. Applying this method to the loop reconstruction test increases the error by a small amount. Overall, the error in the phosphorus position increased by about 1 Å, and the backbone RMSD for the activation loop increased an insignificant amount (Figure 5 and Table 1). We note that Figure 5A clearly shows two major “basins” of attraction for the phosphorylated amino acid. The one with the lower energy places the phosphate in contact with the Arg cluster; the higher energy state places the phosphate in solution. These two conformations, which we will call “active” and “inactive,” respectively, will be examined further below.

When predicting the structural change upon phosphorylation of CDK2, this strategy permits Tyr179, as well as the three arginine residues that form salt bridges with pThr160, to re-optimize around the loop. The prediction with the simultaneous optimization of the loop and surrounding side chains results in a phosphorous atom error of 0.8 Å and a backbone RMSD of 2.9 Å (Figures 3 and 5B, Table 1). The phosphorus atom position is quite accurate, whereas the remainder of the loop may require the optimization of other structural elements to assume the active conformation. The prediction clearly captures the qualitative change in conformation upon phosphorylation, however. Additionally, we performed the above calculations again, except this time substituting the three Arg residues of the arginine cluster with Lys residues (unpublished results). In this case, the phosphate does not localize to the new “Lys cluster,” and we predict that the kinase would not be active. The preference of phosphorylated side chains for interacting with Arg, relative to Lys, has been noted previously, and we will explore this issue further in a separate publication.

### CDK2 Phosphorylation in the Absence of Cyclin Binding

Cyclin A binding is required for the full activation of CDK2 because phosphorylated CDK2 without cyclin A exhibits only 0.3% of the activity of the fully active, phosphorylated, cyclin A–bound structure [3]. The crystal structure of phosphorylated CDK2 in the absence of cyclin A shows that the activation loop is disordered [45]. The loop thus likely adopts several dynamically interconverting conformations with the pThr fully solvated, instead of bound by the arginine cluster. The activation loop in unphosphorylated CDK2 in the absence of cyclin A is ordered, albeit with somewhat elevated B-factors (roughly 60).

**Figure 6:**
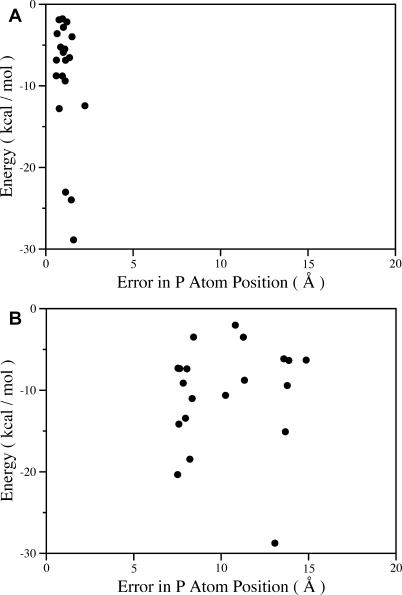
Differences in Conformational Predictions for Phosphorylated CDK2 with and without Cyclin A Bound The 20 lowest-energy loops are considered for predicting the conformation of residues 152–163 upon in silico phosphorylation of Thr160 (A) in cyclin-bound CDK2 and (B) CDK2 in the absence of cyclin A. In (B), the *x*-axis represents the deviation of the phosphate from its position in the fully activated CDK2/cyclin A complex; the activation loop and pThr160 are disordered in the crystal structure of phosphorylated CDK2 in the absence of cyclin A. These calculations were performed with a consistent set of parameters that do not bias the results toward well-ordered loop structures. In the absence of cyclin A, the phosphate does not localize to the Arg cluster as in the cyclin bound case, and the 20 lowest-energy structures show considerable diversity in conformation.

The results of predicting the loop in the phosphorylated, cyclin A–unbound structure [45], showed that the phosphate did not localize to the Arg cluster, which is qualitatively consistent with phosphorylation not contributing to activation (results not shown). However, the low-energy predicted loop conformations showed the phosphate localized at a different position, and the results did not provide any evidence that the loop should in fact be disordered. This is due to a deliberate bias in the loop prediction algorithm, which was originally designed to predict well-structured loops, not ensembles of structures characterizing a disordered loop. A full solution will require an algorithm that obeys detailed balance and thus correctly treats the loop conformational entropy; we have recently developed a Monte Carlo version of the loop sampling algorithm that accomplishes this goal, and we will report results in due course. As a preliminary step, we have performed the calculations with one key modification to the existing loop prediction algorithm. Specifically, the algorithm constrains the loop conformations to be relatively close to the body of the protein using a simple distance cutoff; more than 99% of loops in the Protein Data Bank (PDB) satisfy this criterion [40]. However, this restriction prevents disordered loops from exploring conformations in which the loop is well solvated and makes few contacts to the remainder of the protein. By simply turning off this screening, the loop prediction for phosphorylated CDK2 in the absence of cyclin A does show evidence of diverse low-energy conformations (Figure 6B), in the sense that the 20 lowest-energy conformations are highly diverse; the phosphorylated Thr160 is well solvated in most of these. Turning off the screening does not significantly affect the results for the in silico phosphorylation of CDK2 with cyclin A bound: pThr160 still localizes strongly to the Arg cluster (Figure 6A) in all 20 of the lowest-energy conformations. Thus, in this case, the computer simulations can provide at least a qualitative prediction that phosphorylation of CDK2 in the absence of cyclin binding leads to disorder. More work is clearly required to test this capability in other cases of phosphorylation-induced disorder; the remainder of our results focuses exclusively on cases in which the phosphorylated loops are well ordered.

### Substitution of Glu for the Phosphorylated Thr in the Activation Loop of CDK2

**Figure 7:**
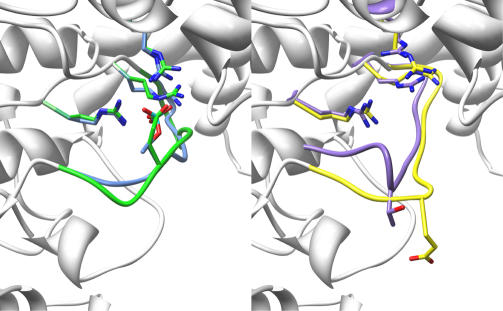
Active and Inactive Conformations of the CDK2 Activation Loop Left: Blue represents the crystal structures of the phospho-CDK2/cyclin A complex, and green represents the T160E-predicted active-like conformation. The Arg cluster is shown in stick representation. The carboxylate group of Glu160 and the phosphate group of pThr160 are almost exactly superimposed. Right: Purple represents the crystal structure of unphosphorylated CDK2/cyclin A, and yellow represents the predicted inactive conformation of T160E. These two structures are qualitatively similar in that Thr160 and Glu160 both point out into solvent and the Arg cluster is better solvated. Figures prepared with Chimera [72].

Among proteins that require phosphorylation for activation, there are many examples of a constitutively active mutant in which the phosphorylatable residue is substituted with either aspartate or glutamate [51–55]. Connell-Crowley et al., however, show that in the CDK2/cyclin A complex, a T160E mutation confers no additional activity to the complex [47]. To examine why, we use the active form of CDK2/cyclin A to create a CDK2/cyclin A T160E mutant in silico, and then use our prediction protocol to predict the conformation of the activation loop, residues 152–163 in the mutant protein. Using the protocol in which side chains surrounding the loop are optimized concurrently with the loop itself, the lowest-energy structure places the Cβ atom of Glu160 9 Å away from its position in the wild-type active loop (Figure 7). The side chain of Glu160 is fully solvated, and the overall loop conformation is reminiscent of the “inactive” conformations identified in the loop prediction results with pThr160. On the other hand, the second lowest-energy conformation places the side chain of Glu160 in a position that is highly analogous to the position of pThr160 in the CDK2/cyclin A complex (i.e., contacting the Arg cluster; see Figure 7). We refer to this as the “active” conformation.

As shown in Table 2, the energy difference between these two states is small for the T160E mutant, with the inactive conformation slightly more stable than the active conformation. For the pThr160 structure, as discussed above, the active structure is correctly predicted to be lower in energy, and the energy gap is somewhat larger (18 kcal/mol). These relatively small differences in overall energies of the active and inactive conformations mask very large differences in the individual energy components. Specifically, the active conformation is strongly favored by the Coulomb electrostatics term in the molecular mechanics energy (172 kcal/mol for the Glu160 case and 298 kcal/mol for the pThr160 case), primarily due to the interaction of the negatively charged side chain of Glu160 or pThr160 with the Arg cluster. On the other hand, the solvation free energy computed from the Generalized Born solvent model strongly favors the inactive conformations, primarily because both Glu160/pThr160 and the Arg cluster are much more solvent exposed in this conformation. The covalent term favors the inactive form; in other words, the active form involves significant internal strain which is relieved in the more open inactive state. In the Glu160 case, the energy differences in the Coulomb and solvation terms largely cancel, and the inactive state winds up slightly lower in energy. In the pThr160 case, the difference in the Coulomb term is significantly larger than the solvation term, causing the active state to be favored by a significant margin.

**Table 2:**
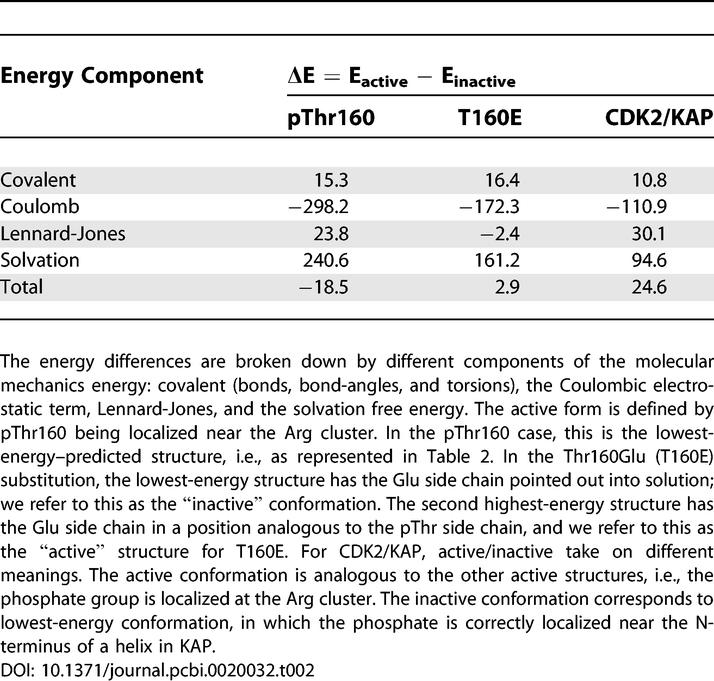
Energy Differences (kcal/mol) between “Active” and “Inactive” Conformations of the Activation Loop

The differences between Glu160 and pThr160 of course derive primarily from the difference in the total charge on the side chains. The −2 charge on pThr nearly doubles the favorable Coulombic electrostatic attraction to the Arg cluster relative to the singly charged carboxylate group on Glu. The more highly charged pThr side chain also has a more favorable solvation free energy in the inactive form, but the overall difference in solvation free energy does not change by as great a factor, in part because greater solvation of the Arg cluster in the inactive state represents a significant contribution to the difference in solvation free energy, and this component is essentially independent of whether Glu or pThr is present at residue 160.

### Phosphorylated CDK2 in Complex with Its Phosphatase

Finally, we examine conformational changes in the activation loop necessary for dephosphorylation to occur. KAP is responsible for the dephosphorylation, and subsequent inactivation, of CDK2 [3,46]. A crystal structure of the CDK2/KAP complex shows that pThr160 localizes to the N-terminus of a helix in the KAP protein, and interacts in that position with a single arginine side chain. The phosphate group must be stable enough in this position to allow the phosphatase to “pull” pThr160 from the Arg cluster on CDK2. Given the highly favorable electrostatic interactions between pThr160 and the three Arg side chains in the cluster, this did not seem intuitively obvious.

Nonetheless, the loop predictions in the CDK2/KAP complex did in fact place the phosphorous atom within 0.5 Å of its position in the crystal structure (Table 1); the lowest energy structure with pThr160 placed in contact with the Arg cluster is approximately 25 kcal/mol higher in energy. Table 2 helps to explain why. The phosphate placed close to the Arg cluster does in fact have much more favorable electrostatic interactions, as measured by the Coulombic term in the molecular mechanics energy function (111 kcal/mol difference). However, the solvation term strongly favors the pThr160 in its correct position in contact with KAP, due largely to the improved solvent accessibility of the Arg cluster after the pThr160 is removed from contact. In addition, the pThr160 is not as buried in the KAP complex. Finally, the covalent terms again indicate significantly higher internal strain in the activation loop when the pThr160 is in contact with the Arg cluster, and much of this strain is relieved when the loop adopts the phosphatase-bound conformation (this might be referred to as a spring-loaded mechanism).

### Predicting Conformational Changes of Loops in Response to Phosphorylation

Encouraged by our results on CDK2, we have performed similar calculations on a larger, more diverse test set (Table 3). Our results show that in all cases of loop reconstruction, we reproduce the structure of the phosphorylated loop with high accuracy (Table 4). We reconstruct seven of the loops to within 1 Å backbone RMSD with respect to the original crystal structure and predict the phosphorus atom to within 0.5 Å of its crystallographic position. Although our predictions are not error free, the relatively small magnitude of the error gives us some confidence that we can predict the correct conformation of a phosphorylated loop using our hierarchical sampling methodology, all-atom force field, and Generalized Born solvation model.

**Table 3:**
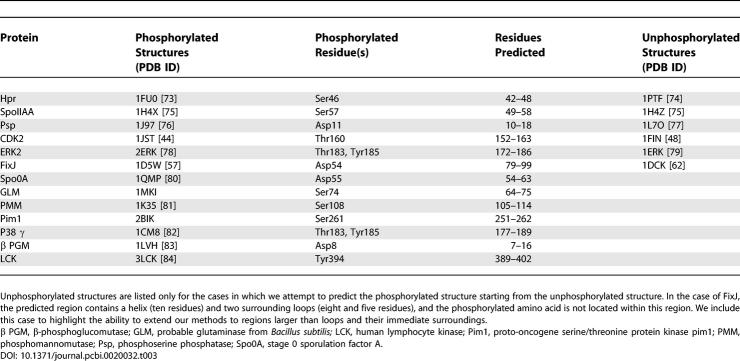
Proteins Used in Our Test Set

**Table 4:**
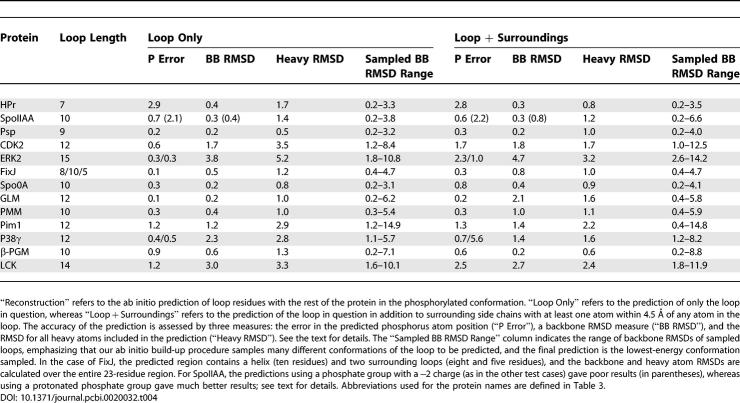
Reconstruction of Phosphorylated Loop Conformations

In cases in which suitable unphosphorylated and phosphorylated structures of the same protein exist, we sought to extend this methodology to the more realistic situation in which we wish to predict the phosphorylated structure from the unphosphorylated structure. In these cases, we start from the crystal structure of the unphosphorylated protein, phosphorylate the residue of interest in silico, and predict the structure of the phosphorylated loop and its surroundings. We refer to this test as “loop prediction.” In this test, sampling only the conformation of the loop itself (Table 5, “loop only”) is successful in some cases, such as histidine-containing phosphocarrier protein (HPr), in which the conformational changes upon phosphorylation are small. In other cases, this strategy performs poorly because the phosphorylation induces substantial conformational changes in the surroundings of the loop, as discussed for CDK2 above. In order to capture these essential structural rearrangements in the region surrounding the loop, we also optimize the conformations of all side chains that have at least one atom within 4.5 Å of the loop, as described in Materials and Methods. This method permits all cases, excepting extracellular signal-regulated kinase 2 (ERK2), to be predicted with near-atomic accuracy, especially the phosphate group itself. Encouragingly, our molecular mechanics methods appear to be capable of not only predicting when significant conformational changes occur, as in CDK2, but also when phosphorylation induces little conformational change, as in HPr.

**Table 5:**
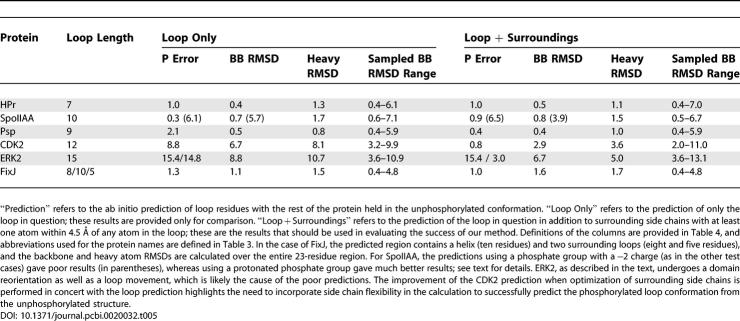
Prediction of Phosphorylated Loop Conformations

ERK2 is unsuccessful because our existing sampling methods do not permit us to sample global structural changes like the changes in domain orientation that occur upon dual phosphorylation of ERK2 [3]. Given that we can accurately reconstruct the active conformation of the ERK2 activation loop, this inability to account for the domain reorientation is likely the cause of the poor prediction of the activation loop structure. This test case highlights a fundamental limitation of our current prediction strategy; the only solution is to expand the sampling to explicitly sample domain orientations, either in a manner analogous to our helix prediction methods or by using normal modes or other methods capable of sampling low-frequency, global motions of the protein.

The SpoIIAA case also requires explanation. The initial loop prediction and reconstruction tests on this case, using an unprotonated (−2) phosphate group as in the other cases, gave very poor results, listed in parentheses in Tables 4 and 5. Examining the structure of the phosphorylated protein suggested a possible explanation. The pSer58 side chain lies within approximately 5 Å of the side chain of Asp56. The pKa of the phosphorylated amino acids is approximately 6, which implies that the predominant charge state is generally −2 under physiological conditions, and the good results we obtain for the other test cases using a −2 phosphate seem to confirm this assumption. However, it is well known that the pKa's of titratable groups can be significantly shifted in macromolecules due to desolvation and/or the electrostatic field from the rest of the protein. In this case, the close proximity of Asp56 is likely to shift the pKa higher and favor the −1, protonated state of the phosphate group on pSer58 (the pH used in the crystallization conditions is 6.5). In fact, performing the loop predictions with a protonated phosphate group leads to excellent results. In current work, we are implementing a new version of our molecular mechanics algorithms that allows automatic identification of the optimal protonation state for phosphorylated amino acids and other titratable groups (especially His), using a thermodynamic cycle analogous to that employed by the numerous Poisson-Boltzmann based pKa prediction algorithms [56].

### Predicting Conformational Changes of Helices in Response to Phosphorylation

To test our ability to model conformational responses that extend beyond a single loop and its surroundings, we consider the repacking of helices that is known to occur upon phosphorylation of response regulator proteins of prokaryotes [3]. The response regulator proteins are the second part of the two-component signaling system that bacteria use to sense and respond to their environment. In most two-component systems, a sensor histidine kinase autophosphorylates a His upon sensing signal and transfers this phosphate to an Asp on a response regulator. Upon phosphorylation, certain structural changes occur, most notably a helix shift and loop rearrangement, that ultimately allow the response regulator to activate transcription of a set of genes.

**Figure 8:**
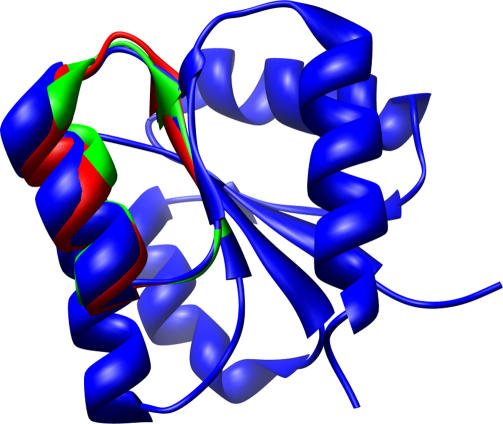
Helix Reconstruction and Prediction of FixJ The loop-helix-loop region of FixJ was predicted starting from either the phosphorylated structure (blue) or the unphosphorylated structure (unpublished results). The reconstruction (starting from the phosphorylated structure) is in red, and the prediction (starting from the unphosphorylated structure) is in green. Figure prepared with Chimera [72].

We used our existing helix prediction algorithm to model the structural changes of the response regulator protein FixJ (from Sinorhizobium meliloti [57]), specifically, the loop-helix-loop region that undergoes a helix shift and loop rearrangements. Another response regulator protein, Spo0A, has crystal structures in both the phosphorylated and unphosphorylated forms, but the crystal structure for the unphosphorylated form of Spo0A is a domain-swapped dimer, solved at a pH of 4.5. As discussed in Materials and Methods, the helix prediction algorithm employs rigid body sampling for the helix (residues 87–94) combined with the hierarchical loop sampling algorithm for predicting the connecting loops [42] (residues 79–86 and 95–99). The overall backbone RMSD for reconstructing this region in the phosphorylated crystal structure is 0.5 Å, with most of the error arising from the first flanking loop, which is longer, closest to the phosphate, and more exposed to solvent (Table 4 and Figure 8). The results from in silico phosphorylation of the unphosphorylated structure are also quite good, with an overall RMSD of 1.6 Å.

### Conclusion

We have described our initial efforts to predict and understand how the structure of a protein is modulated by post-translational phosphorylation. We believe that this work has practical significance in that we demonstrate that it is possible to make testable predictions concerning the structure of phosphorylated proteins, given the structure of the unphosphorylated protein and a known site of phosphorylation. In this work, we have restricted our efforts to predicting relatively modest, localized conformational changes, and we have assumed knowledge of which portions of the protein undergo significant conformational change (those portions closest to the phosphorylated amino acid). Despite these limitations, this modeling technology can be used to create hypotheses about mechanisms of regulation by phosphorylation that can then be tested experimentally, e.g., by site-directed mutagenesis. Applications of this type are underway. In addition, our in-depth case study of CDK2 illustrates how the computational methods can be used to obtain new insights into the energetic underpinnings of phosphorylation-induced conformational change even in a well-studied system.

## Materials and Methods

### Dataset selection.

We searched the PDB [58] for phosphorylated protein structures determined by X-ray crystallography (with better than 2.5 Å resolution) that are phosphorylated on well-ordered loop structures that were less than 15 residues in length. We exclude structures in which phosphorylation causes a large, global rearrangement of the protein structure, such as a hinge-bending movement or domain rearrangement, as is the case with glycogen phosphorylase [59] and insulin receptor tyrosine kinase [60,61]. In order to test the limitations of our method, we include one test case, ERK2, in which phosphorylation causes a domain rearrangement [3]. We also include a prokaryotic response regulator, FixJ, in which phosphorylation of an Asp induces a significant conformational change in the orientation of a helix [57,62]; this case is successfully treated with an extension of our methods described below. The test set is listed in Table 3.

We determined the loop length that we would predict for each phosphorylated structure using visual inspection. In the cases in which crystallographic structures are available for both the unphosphorylated and phosphorylated protein, these structures were superimposed and the residues to be predicted were defined as the portion of the loop that deviated in the superposition. For the reconstruction of phosphorylated structures without knowledge of the unphosphorylated form, the loop residues to be optimized were determined using a combination of visual inspection of secondary structure and crystallographic B-factors.

### Molecular mechanics energy function.

All energy calculations use the OPLS-AA force field [37,63,64] and the Surface Generalized Born (SGB) model of solvation [49,50]. The molecular mechanics energy function represents electrostatics by a relatively simple model of fixed atomic partial charges interacting through the Coulomb approximation. The solvent model captures key effects of desolvation with relatively modest computational expense. Despite the simplicity of the energy function (i.e., it neglects polarizability contributions to electrostatics, and implicit solvent models have well-known limitations), it performs well in predicting conformations of phosphorylated loops (see Results).

The force field parameters for the phosphorylated amino acids were generated by an automated atom-typing algorithm provided in the Impact software package. The atomic partial charges for the phosphorylated amino acid side chains were adjusted slightly from the default values by performing quantum chemistry calculations. The partial charges for phosphoserine (pSer) and phosphothreonine (pThr) were taken from previous work by Wong et al. [65], whereas charges for phosphotyrosine (pTyr) and phosphoaspartate (pAsp) were determined in their −2 and −1 charge states by performing quantum mechanical calculations with the software program Jaguar [66]. Methyl-benzyl-phosphate was used to represent the pTyr side chain, and acetyl phosphate was used for pAsp. Geometry optimization of the phosphate ion was carried out at the HF/6–31G** level, incorporating a condensed-phase environment via a self-consistent reaction field (SCRF) algorithm [67,68]. Single point calculations were performed at the LMP2/cc-pvtz(-f) level, also with SCRF treatment of solvation. Electrostatic potential fitting was used to determine the partial charges. The atomic partial charges for all four phosphorylated amino acids are provided in Tables S1–S4.

### Loop prediction methodology.

This study uses the method of Jacobson et al. [40] for predicting loop conformations. In brief, the loop prediction methodology uses an ab initio dihedral sampling scheme to enumerate conformations of the loop backbone that are free from steric clashes. Other methods have employed similar dihedral angle sampling schemes, including ICM [69], CONGEN [70], and the work of DePristo, et al [71]. Unlike Monte Carlo and Molecular Dynamics sampling schemes, the algorithm itself has no knowledge of the starting conformation of the loop, and therefore, does not start predictions based on a starting structure of the loop. These closed backbone conformations are clustered, and a single member of each cluster is then selected for side chain addition and optimization, followed by complete energy minimization. The lowest energy structure is selected as the output of the loop prediction algorithm. The algorithm also permits explicit treatment of crystal packing. We have used this capability in this work (in the “loop reconstruction” cases, as described below), but did not identify any clear crystal-packing artifacts relevant to the conformations of the phosphorylated loops.

The Jacobson et al. paper [40] describes a hierarchical refinement procedure in which multiple iterations of the loop prediction algorithm are used to reduce errors caused by insufficient sampling. The parameters in this scheme have been slightly modified in this study due to the inclusion of some rather long loops (up to 15 residues) in our test set. The first stage allows for unrestrained sampling. After this stage is complete, the top ten lowest energy structures are passed to a first refinement stage in which more extensive sampling is performed around these low-energy basins. Specifically, loop conformations in this stage are only retained if all of the Cα atoms in the loop are within 10 Å of the starting loop structure (which is one of the ten lowest-energy structures from the initial stage). The five resulting lowest-energy structures from this stage are subjected to a second round of refinement, in which the maximum Cα deviation is restricted to 5 Å. Finally, the five lowest-energy structures from this stage are subjected to a third and final round of refinement, in which the maximum Cα deviation is restricted to 2.5 Å. In all, this procedure provides a rank-ordered list consisting of about 250 loops and their associated energies in the context of the full protein. The final prediction is the loop conformation with the lowest energy.

Most published tests of loop prediction methods, including the Jacobson et al. paper [40], evaluate their success by the ability to reconstruct loops in a native protein structure. In these tests, all portions of the protein other than the loop in question remain in the native conformation during the simulation. Success of a prediction methodology in such a test is an important prerequisite for more realistic applications. We perform loop reconstruction tests in this work to assess the ability of the molecular mechanics energy function to identify correct conformations of phosphorylated loops, and to ensure that the sampling methods are sufficient to generate near-native conformations. However, predicting conformational changes induced by phosphorylation, i.e., by phosphorylating a protein in silico, is qualitatively more challenging. In the cases we consider, the sites of phosphorylation are located on loops, and most of the conformational change is localized to that loop. However, there is always some degree of conformational rearrangement in the vicinity of the loop, especially in the conformations of side chains contacting it. Similarly, predicting the conformation of a loop in a homology model is more challenging than reconstructing a loop in a native protein structure because the environment surrounding any given loop in a homology model contains errors that can affect the loop prediction accuracy. We address this issue by performing rotamer optimization and minimization of side chains in the immediate vicinity of the loop concurrently with the optimization of the side chains on the loop itself. In the test set presented here, this strategy performs well in predicting local structural changes, despite the fact that there are also some changes in backbone conformation in the surroundings. We speculate that small changes in the conformations of the surrounding side chains can compensate for not explicitly allowing backbone relaxation. We also use this strategy in the control studies of reconstructing phosphorylated loops in which, interestingly, it sometimes improves the accuracy.

### Helix prediction methodology.

The helix prediction algorithm used in this paper is based on the work of Li et al [42]. Briefly, the helix backbone is treated as a rigid body and sampled in six degrees of freedom (three translations and three rotations), and the two flanking loops are sampled using the loop prediction algorithm described above. Again, the method broadly samples the possible configurations of the helix and surrounding loops, independent of any starting configuration, and then hierarchically samples more finely around low-energy basins. As with the loop prediction algorithm, side chains on the loop-helix-loop region, and the surroundings if desired, are sampled using a rotamer-based optimization algorithm.

### Development of “rotamer” libraries for phosphorylated residues.

The loop and helix prediction algorithms require the use of a rotamer library for sampling side chain conformations. However, the number of phosphorylated residues in the PDB is insufficient to construct these libraries by statistical analysis of observed side chain conformations. Instead, we obtained a rotamer library by exhaustively exploring the energy landscape of the side chains of phosphorylated amino acid dipeptides, and retaining all conformations below an energy threshold that ensures inclusion of all experimentally observed rotamers. We use the same energy function as in the rest of the work, i.e., OPLS-AA/SGB. The pSer and pThr side chains are sampled at 10° resolution, whereas pTyr and pAsp are sampled at 30° resolution, due to the larger number of rotatable bonds. The total number of rotamers are 802 for pSer, 501 for pThr, 858 for pTyr, and 1,008 for pAsp. These values do not include the rotation of the phosphate group about the phosphoester bond, which is sampled uniformly at the same resolution as the other bonds. The rotamer libraries are provided in Tables S5–S8.

## Supporting Information

Table S1 Partial Atomic Charges Employed for pThr (38 KB DOC)

Table S2 Partial Atomic Charges Employed for pSer (35 KB DOC)

Table S3 Partial Atomic Charges Employed for pTyr (44 KB DOC)

Table S4 Partial Atomic Charges Employed for pAsp (37 KB DOC)

Table S5 Rotamer Library Used for Sampling pThr (54 KB DOC)

Table S6 Rotamer Library Used for Sampling pSer (74 KB DOC)

Table S7 Rotamer Library Used for Sampling pTyr (77 KB DOC)

Table S8 Rotamer Library Used for Sampling pAsp (85 KB DOC)

### Accession Numbers

The Protein Data Bank (http://www.rcsb.org/pdb) accession numbers for the proteins discussed in this paper are as follows: β PGM, (PDB ID: 1LVH); CDK2, phosphorylated (PDB ID: 1JST); CDK2, unphosphorylated (PDB ID: 1FIN); CDK2/KAP complex (PDB ID: 1FQ1); cyclin A (PDB ID: 1B39); ERK2, phosphorylated (PDB ID: 2ERK); ERK2, unphosphorylated (PDB ID: 1ERK); FixJ, phosphorylated (PDB ID: 1D5W); FixJ, unphosphorylated (PDB ID: 1DCK); GLM, (PDB ID: 1MKI); Hpr, phosphorylated (PDB ID: 1FU0); Hpr, unphosphorylated (PDB ID: 1PTF); LCK, (PDB ID: 3LCK); Pim1, (PDB ID: 2BIK); PMM, (PDB ID: 1K35); Psp, phosphorylated (PDB ID: 1J97); Psp, unphosphorylated (PDB ID: 1L7O); P38 γ, (PDB ID: 1CM8); SpoIIAA, phosphorylated (PDB ID: 1H4X); and SpoIIAA, unphosphorylated (PDB ID: 1H4Z); Spo0A, (PDB ID: 1QMP).

## Abbreviations

CDK2: cyclin dependent kinase 2
ERK2: extracellular signal-regulated kinase 2
HPr: histidine-containing phosphocarrier protein
KAP: kinase-associated phosphatase
PDB: Protein Data Bank
RMSD: root mean square deviation
