# Supplementary material for: Conformational Changes in Protein Loops and Helices Induced by Post-Translational Phosphorylation
Source: PLoS Comput Biol. 2006 Apr 21;2(4):e32. doi: 10.1371/journal.pcbi.0020032 (PMC1440919; doi:10.1371/journal.pcbi.0020032)
Supplement: Supplementary file 2 [file pcbi.0020032.st002.doc]

Conformational Changes in Protein Loops and Helices Induced by Post-Translational Phosphorylation

E. S. Groban, A. Narayanan, and M. P. Jacobson

**Supporting Information**

**Supplementary Table 2**: Partial atomic charges employed for pSer.

| Phosphorylated Serine | | |
| --- | --- | --- |
| Atom ID | Charge in -2 state | Charge in -1 state |
| CB | 0.280 | 0.280 |
| 1HB | -0.030 | 0.035 |
| 2HB | -0.030 | 0.035 |
| OG | -0.700 | -0.600 |
| P | 1.570 | 1.500 |
| O1P | -1.030 | -1.000 |
| O2P | -1.030 | -1.000 |
| O3P | -1.030 | -0.750 |
| HO | NA | 0.500 |
