# Supplementary material for: Conformational Changes in Protein Loops and Helices Induced by Post-Translational Phosphorylation
Source: PLoS Comput Biol. 2006 Apr 21;2(4):e32. doi: 10.1371/journal.pcbi.0020032 (PMC1440919; doi:10.1371/journal.pcbi.0020032)
Supplement: Supplementary file 3 [file pcbi.0020032.st003.doc]

Conformational Changes in Protein Loops and Helices Induced by Post-Translational Phosphorylation

E. S. Groban, A. Narayanan, and M. P. Jacobson

**Supporting Information**

**Supplementary Table 3**: Partial atomic charges employed for pTyr.

| Phosphorylated Tyrosine | | |
| --- | --- | --- |
| Atom ID | Charge in -2 state | Charge in -1 state |
| CB | -0.005 | -0.005 |
| 1HB | 0.060 | 0.060 |
| 2HB | 0.060 | 0.060 |
| CG | -0.115 | -0.115 |
| CD1 | -0.115 | -0.115 |
| HD1 | 0.115 | 0.115 |
| CE1 | -0.115 | -0.115 |
| HE1 | 0.115 | 0.115 |
| CZ | 0.220 | 0.240 |
| CE2 | -0.115 | -0.115 |
| HE2 | 0.115 | 0.115 |
| CD2 | -0.115 | -0.115 |
| HD2 | 0.115 | 0.115 |
| OH | -0.700 | -0.600 |
| P | 1.570 | 1.550 |
| O1P | -1.030 | -0.970 |
| O2P | -1.030 | -0.970 |
| O3P | -1.030 | -0.750 |
| HO | NA | 0.500 |
