# Supplementary material for: Conformational Changes in Protein Loops and Helices Induced by Post-Translational Phosphorylation
Source: PLoS Comput Biol. 2006 Apr 21;2(4):e32. doi: 10.1371/journal.pcbi.0020032 (PMC1440919; doi:10.1371/journal.pcbi.0020032)
Supplement: Supplementary file 4 [file pcbi.0020032.st004.doc]

Conformational Changes in Protein Loops and Helices Induced by Post-Translational Phosphorylation

E. S. Groban, A. Narayanan, and M. P. Jacobson

**Supporting Information**

**Supplementary Table 4**: Partial atomic charges employed for pAsp.

| Phosphorylated Aspartate | | |
| --- | --- | --- |
| Atom ID | Charge in -2 state | Charge in -1 state |
| CB | -0.470 | -0.440 |
| 1HB | 0.220 | 0.230 |
| 2HB | 0.220 | 0.230 |
| CG | 0.940 | 0.870 |
| OD1 | -0.590 | -0.540 |
| OD2 | -0.730 | -0.670 |
| P | 1.470 | 1.500 |
| O1P | -1.020 | -0.950 |
| O2P | -1.020 | -0.950 |
| O3P | -1.020 | -0.790 |
| HO | NA | 0.510 |
