# Supplementary material for: Conformational Changes in Protein Loops and Helices Induced by Post-Translational Phosphorylation
Source: PLoS Comput Biol. 2006 Apr 21;2(4):e32. doi: 10.1371/journal.pcbi.0020032 (PMC1440919; doi:10.1371/journal.pcbi.0020032)
Supplement: Supplementary file 5 [file pcbi.0020032.st005.doc]

Conformational Changes in Protein Loops and Helices Induced by Post-Translational Phosphorylation

E. S. Groban, A. Narayanan, and M. P. Jacobson

**Supporting Information**

**Supplementary Table 5**: Rotamer library used for sampling pThr. Numbers should be multiplied by 10 to obtain the torsion angle in degrees. The columns represent the sampled heavy-atom torsion angles, other than the rotation about the O-P bond in the phosphate group, which is sampled uniformly. χ_1_ represents rotation about the C_α_-C_β_ bond, and χ_2_ is the rotation about the C_β_-O_γ_ bond.

```
-18  -18
-18  6
-18  7
-18  8
-18  9
-18  10
-18  11
-18  12
-18  13
-18  14
-18  15
-18  16
-18  17
-18  18
-17  -18
-17  6
-17  7
-17  8
-17  9
-17  10
-17  11
-17  12
-17  13
-17  14
-17  15
-17  16
-17  17
-17  18
-16  -18
-16  6
-16  7
-16  8
-16  9
-16  10
-16  11
-16  12
-16  13
-16  14
-16  15
-16  16
-16  17
-16  18
-15  -18
-15  6
-15  7
-15  8
-15  9
-15  10
-15  11
-15  12
-15  13
-15  14
-15  15
-15  16
-15  17
-15  18
-14  -18
-14  5
-14  6
-14  7
-14  8
-14  9
-14  10
-14  11
-14  12
-14  13
-14  14
-14  15
-14  16
-14  17
-14  18
-13  -18
-13  5
-13  6
-13  7
-13  8
-13  9
-13  10
-13  11
-13  12
-13  13
-13  14
-13  15
-13  16
-13  17
-13  18
-12  -18
-12  5
-12  6
-12  7
-12  8
-12  9
-12  10
-12  11
-12  12
-12  13
-12  14
-12  15
-12  16
-12  17
-12  18
-11  -18
-11  -6
-11  -5
-11  6
-11  7
-11  8
-11  9
-11  10
-11  11
-11  12
-11  13
-11  14
-11  15
-11  16
-11  17
-11  18
-10  -18
-10  -6
-10  7
-10  8
-10  9
-10  10
-10  11
-10  12
-10  13
-10  14
-10  15
-10  16
-10  17
-10  18
-9  -18
-9  -6
-9  7
-9  8
-9  9
-9  10
-9  11
-9  12
-9  13
-9  14
-9  15
-9  16
-9  17
-9  18
-8  -18
-8  -6
-8  8
-8  9
-8  10
-8  11
-8  12
-8  13
-8  14
-8  15
-8  16
-8  17
-8  18
-7  -18
-7  -6
-7  8
-7  9
-7  10
-7  11
-7  12
-7  13
-7  14
-7  15
-7  16
-7  17
-7  18
-6  -18
-6  8
-6  9
-6  10
-6  11
-6  12
-6  13
-6  14
-6  15
-6  16
-6  17
-6  18
-5  -18
-5  8
-5  9
-5  10
-5  11
-5  12
-5  13
-5  14
-5  15
-5  16
-5  17
-5  18
-4  -18
-4  -6
-4  7
-4  8
-4  9
-4  10
-4  11
-4  12
-4  13
-4  14
-4  15
-4  16
-4  17
-4  18
-3  -18
-3  -6
-3  -5
-3  7
-3  8
-3  9
-3  10
-3  11
-3  12
-3  13
-3  14
-3  15
-3  16
-3  17
-3  18
-2  -18
-2  -6
-2  -5
-2  7
-2  8
-2  9
-2  10
-2  11
-2  12
-2  13
-2  14
-2  15
-2  16
-2  17
-2  18
-1  -18
-1  -6
-1  6
-1  7
-1  8
-1  9
-1  10
-1  11
-1  12
-1  13
-1  14
-1  15
-1  16
-1  17
-1  18
0  -18
0  -6
0  6
0  7
0  8
0  9
0  10
0  11
0  12
0  13
0  14
0  15
0  16
0  17
0  18
1  -18
1  7
1  8
1  9
1  10
1  11
1  12
1  13
1  14
1  15
1  16
1  17
1  18
2  -18
2  7
2  8
2  9
2  10
2  11
2  12
2  13
2  14
2  15
2  16
2  17
2  18
3  -18
3  8
3  9
3  10
3  11
3  12
3  13
3  14
3  15
3  16
3  17
3  18
4  -18
4  8
4  9
4  10
4  11
4  12
4  13
4  14
4  15
4  16
4  17
4  18
5  -18
5  8
5  9
5  10
5  11
5  12
5  13
5  14
5  15
5  16
5  17
5  18
6  -18
6  8
6  9
6  10
6  11
6  12
6  13
6  14
6  15
6  16
6  17
6  18
7  -18
7  8
7  9
7  10
7  11
7  12
7  13
7  14
7  15
7  16
7  17
7  18
8  -18
8  8
8  9
8  10
8  11
8  12
8  13
8  14
8  15
8  16
8  17
8  18
9  -18
9  8
9  9
9  10
9  11
9  12
9  13
9  14
9  15
9  16
9  17
9  18
10  -18
10  8
10  9
10  10
10  11
10  12
10  13
10  14
10  15
10  16
10  17
10  18
11  -18
11  7
11  8
11  9
11  10
11  11
11  12
11  13
11  14
11  15
11  16
11  17
11  18
12  -18
12  7
12  8
12  9
12  10
12  11
12  12
12  13
12  14
12  15
12  16
12  17
12  18
13  -18
13  6
13  7
13  8
13  9
13  10
13  11
13  12
13  13
13  14
13  15
13  16
13  17
13  18
14  -18
14  6
14  7
14  8
14  9
14  10
14  11
14  12
14  13
14  14
14  15
14  16
14  17
14  18
15  -18
15  6
15  7
15  8
15  9
15  10
15  11
15  12
15  13
15  14
15  15
15  16
15  17
15  18
16  -18
16  6
16  7
16  8
16  9
16  10
16  11
16  12
16  13
16  14
16  15
16  16
16  17
16  18
17  -18
17  6
17  7
17  8
17  9
17  10
17  11
17  12
17  13
17  14
17  15
17  16
17  17
17  18
18  -18
18  6
18  7
18  8
18  9
18  10
18  11
18  12
18  13
18  14
18  15
18  16
18  17
```

18 18
