# Supplementary material for: Conformational Changes in Protein Loops and Helices Induced by Post-Translational Phosphorylation
Source: PLoS Comput Biol. 2006 Apr 21;2(4):e32. doi: 10.1371/journal.pcbi.0020032 (PMC1440919; doi:10.1371/journal.pcbi.0020032)
Supplement: Supplementary file 6 [file pcbi.0020032.st006.doc]

Conformational Changes in Protein Loops and Helices Induced by Post-Translational Phosphorylation

E. S. Groban, A. Narayanan, and M. P. Jacobson

**Supporting Information**

**Supplementary Table 6**: Rotamer library used for sampling pSer. Numbers should be multiplied by 10 to obtain the torsion angle in degrees. The columns represent the sampled heavy-atom torsion angles, other than the rotation about the O-P bond in the phosphate group, which is sampled uniformly. χ_1_ represents rotation about the C_α_-C_β_ bond, and χ_2_ is the rotation about the C_β_-O_γ_ bond.

```
-18  -18
-18  -17
-18  -16
-18  -15
-18  -14
-18  -13
-18  -12
-18  -11
-18  -10
-18  7
-18  8
-18  9
-18  10
-18  11
-18  12
-18  13
-18  14
-18  15
-18  16
-18  17
-18  18
-17  -18
-17  -17
-17  -16
-17  -15
-17  -14
-17  -13
-17  -12
-17  -11
-17  -10
-17  -9
-17  7
-17  8
-17  9
-17  10
-17  11
-17  12
-17  13
-17  14
-17  15
-17  16
-17  17
-17  18
-16  -18
-16  -17
-16  -16
-16  -15
-16  -14
-16  -13
-16  -12
-16  -11
-16  -10
-16  -9
-16  7
-16  8
-16  9
-16  10
-16  11
-16  12
-16  13
-16  14
-16  15
-16  16
-16  17
-16  18
-15  -18
-15  -17
-15  -16
-15  -15
-15  -14
-15  -13
-15  -12
-15  -11
-15  -10
-15  -9
-15  6
-15  7
-15  8
-15  9
-15  10
-15  11
-15  12
-15  13
-15  14
-15  15
-15  16
-15  17
-15  18
-14  -18
-14  -17
-14  -16
-14  -15
-14  -14
-14  -13
-14  -12
-14  -11
-14  -10
-14  -9
-14  6
-14  7
-14  8
-14  9
-14  10
-14  11
-14  12
-14  13
-14  14
-14  15
-14  16
-14  17
-14  18
-13  -18
-13  -17
-13  -16
-13  -15
-13  -14
-13  -13
-13  -12
-13  -11
-13  -10
-13  -9
-13  -8
-13  6
-13  7
-13  8
-13  9
-13  10
-13  11
-13  12
-13  13
-13  14
-13  15
-13  16
-13  18
-12  -18
-12  -17
-12  -16
-12  -15
-12  -14
-12  -13
-12  -12
-12  -11
-12  -10
-12  -9
-12  -8
-12  -7
-12  7
-12  8
-12  9
-12  10
-12  11
-12  12
-12  13
-12  14
-12  15
-12  16
-12  17
-12  18
-11  -18
-11  -17
-11  -16
-11  -14
-11  -13
-11  -12
-11  -11
-11  -10
-11  -9
-11  -8
-11  -7
-11  -6
-11  8
-11  9
-11  10
-11  11
-11  12
-11  13
-11  14
-11  15
-11  16
-11  17
-11  18
-10  -18
-10  -17
-10  -16
-10  -15
-10  -14
-10  -13
-10  -12
-10  -11
-10  -10
-10  -9
-10  -8
-10  -7
-10  -6
-10  8
-10  9
-10  10
-10  11
-10  12
-10  13
-10  15
-10  16
-10  17
-10  18
-9  -18
-9  -17
-9  -16
-9  -15
-9  -14
-9  -13
-9  -12
-9  -11
-9  -10
-9  -9
-9  -8
-9  -7
-9  -6
-9  8
-9  9
-9  10
-9  11
-9  12
-9  13
-9  14
-9  15
-9  16
-9  17
-9  18
-8  -18
-8  -17
-8  -16
-8  -15
-8  -14
-8  -13
-8  -12
-8  -11
-8  -10
-8  -9
-8  -8
-8  -7
-8  9
-8  10
-8  11
-8  12
-8  13
-8  14
-8  15
-8  17
-8  18
-7  -18
-7  -16
-7  -15
-7  -14
-7  -12
-7  -11
-7  -10
-7  -9
-7  -8
-7  -7
-7  9
-7  10
-7  11
-7  12
-7  13
-7  14
-7  16
-7  17
-7  18
-6  -18
-6  -17
-6  -16
-6  -15
-6  -14
-6  -13
-6  -12
-6  -11
-6  -10
-6  -9
-6  -8
-6  -7
-6  9
-6  10
-6  11
-6  12
-6  13
-6  14
-6  15
-6  16
-6  17
-6  18
-5  -18
-5  -17
-5  -16
-5  -15
-5  -14
-5  -13
-5  -12
-5  -11
-5  -10
-5  -9
-5  -8
-5  -7
-5  9
-5  10
-5  11
-5  12
-5  13
-5  14
-5  15
-5  16
-5  17
-5  18
-4  -18
-4  -17
-4  -16
-4  -15
-4  -13
-4  -12
-4  -11
-4  -10
-4  -9
-4  -8
-4  -7
-4  8
-4  9
-4  10
-4  11
-4  12
-4  13
-4  14
-4  15
-4  16
-4  17
-4  18
-3  -18
-3  -17
-3  -16
-3  -15
-3  -14
-3  -13
-3  -12
-3  -11
-3  -10
-3  -9
-3  -8
-3  -7
-3  8
-3  9
-3  10
-3  11
-3  12
-3  13
-3  14
-3  15
-3  16
-3  17
-3  18
-2  -18
-2  -17
-2  -16
-2  -15
-2  -14
-2  -13
-2  -12
-2  -10
-2  -9
-2  -8
-2  -7
-2  -6
-2  8
-2  9
-2  10
-2  11
-2  12
-2  13
-2  14
-2  15
-2  16
-2  17
-2  18
-1  -18
-1  -17
-1  -16
-1  -15
-1  -14
-1  -13
-1  -12
-1  -11
-1  -10
-1  -9
-1  -8
-1  -7
-1  8
-1  9
-1  10
-1  11
-1  12
-1  13
-1  14
-1  15
-1  16
-1  17
-1  18
0  -18
0  -17
0  -16
0  -15
0  -14
0  -13
0  -12
0  -11
0  -10
0  -9
0  -8
0  -7
0  7
0  8
0  9
0  10
0  11
0  12
0  13
0  14
0  15
0  16
0  17
0  18
1  -18
1  -17
1  -16
1  -15
1  -14
1  -13
1  -12
1  -11
1  -10
1  -9
1  -8
1  8
1  9
1  10
1  11
1  12
1  13
1  14
1  15
1  16
1  17
1  18
2  -18
2  -17
2  -16
2  -15
2  -14
2  -13
2  -12
2  -11
2  -10
2  -9
2  -8
2  8
2  9
2  10
2  11
2  12
2  13
2  14
2  15
2  16
2  17
2  18
3  -18
3  -17
3  -16
3  -15
3  -14
3  -13
3  -12
3  -11
3  -10
3  -9
3  -8
3  9
3  10
3  11
3  12
3  13
3  14
3  15
3  16
3  17
3  18
4  -18
4  -17
4  -16
4  -15
4  -14
4  -13
4  -12
4  -11
4  -10
4  -9
4  -8
4  9
4  10
4  11
4  12
4  13
4  14
4  15
4  16
4  17
4  18
5  -18
5  -17
5  -16
5  -15
5  -14
5  -13
5  -12
5  -10
5  -9
5  -8
5  9
5  10
5  11
5  12
5  13
5  14
5  15
5  16
5  17
5  18
6  -18
6  -17
6  -16
6  -15
6  -14
6  -13
6  -12
6  -11
6  -10
6  -9
6  9
6  10
6  11
6  12
6  13
6  14
6  15
6  16
6  17
6  18
7  -18
7  -17
7  -16
7  -15
7  -14
7  -13
7  -12
7  -11
7  -10
7  -9
7  9
7  10
7  11
7  12
7  13
7  14
7  15
7  16
7  17
7  18
8  -18
8  -17
8  -16
8  -15
8  -14
8  -13
8  -12
8  -11
8  -10
8  -9
8  9
8  10
8  11
8  12
8  14
8  15
8  16
8  17
8  18
9  -18
9  -17
9  -16
9  -15
9  -14
9  -13
9  -12
9  -11
9  -10
9  -9
9  -8
9  9
9  10
9  11
9  12
9  13
9  14
9  15
9  16
9  17
9  18
10  -18
10  -17
10  -16
10  -15
10  -14
10  -13
10  -12
10  -11
10  -10
10  -9
10  -8
10  9
10  10
10  11
10  12
10  13
10  14
10  15
10  16
10  18
11  -18
11  -17
11  -16
11  -15
11  -14
11  -13
11  -12
11  -11
11  -10
11  -9
11  -8
11  9
11  10
11  11
11  12
11  13
11  14
11  15
11  16
11  17
11  18
12  -18
12  -17
12  -16
12  -15
12  -14
12  -13
12  -12
12  -11
12  -10
12  -9
12  -8
12  8
12  9
12  10
12  11
12  12
12  13
12  14
12  15
12  16
12  17
12  18
13  -18
13  -17
13  -16
13  -15
13  -14
13  -13
13  -12
13  -11
13  -10
13  -9
13  8
13  9
13  10
13  11
13  12
13  13
13  14
13  15
13  16
13  17
13  18
14  -18
14  -17
14  -16
14  -15
14  -14
14  -13
14  -12
14  -11
14  -10
14  -9
14  7
14  8
14  9
14  10
14  12
14  13
14  14
14  15
14  16
14  17
14  18
15  -18
15  -17
15  -16
15  -15
15  -14
15  -13
15  -12
15  -11
15  -10
15  -9
15  8
15  9
15  10
15  11
15  12
15  13
15  14
15  15
15  16
15  17
15  18
16  -18
16  -17
16  -16
16  -15
16  -14
16  -13
16  -12
16  -11
16  -10
16  -9
16  7
16  8
16  9
16  10
16  12
16  13
16  14
16  15
16  16
16  17
16  18
17  -18
17  -17
17  -16
17  -15
17  -14
17  -13
17  -12
17  -11
17  -10
17  -9
17  7
17  8
17  9
17  10
17  11
17  12
17  14
17  15
17  16
17  17
17  18
18  -18
18  -17
18  -16
18  -15
18  -14
18  -13
18  -12
18  -11
18  -10
18  7
18  8
18  9
18  10
18  11
18  12
18  13
18  14
18  15
18  16
18  17
```

18 18
