# Supplementary material for: Conformational Changes in Protein Loops and Helices Induced by Post-Translational Phosphorylation
Source: PLoS Comput Biol. 2006 Apr 21;2(4):e32. doi: 10.1371/journal.pcbi.0020032 (PMC1440919; doi:10.1371/journal.pcbi.0020032)
Supplement: Supplementary file 7 [file pcbi.0020032.st007.doc]

Conformational Changes in Protein Loops and Helices Induced by Post-Translational Phosphorylation

E. S. Groban, A. Narayanan, and M. P. Jacobson

**Supporting Information**

**Supplementary Table 7**: Rotamer library used for sampling pTyr. Numbers should be multiplied by 30 to obtain the torsion angle in degrees. The columns represent the sampled heavy-atom torsion angles, other than the rotation about the O-P bond in the phosphate group, which is sampled uniformly. χ_1_ represents rotation about the C_α_-C_β_ bond, χ_2_ is the rotation about C_β_-C_γ_, and χ_3_ is rotation about the C_ζ_-O bond.

```
-6  -6  -4
-6  -6  -3
-6  -6  -2
-6  -6  2
-6  -6  3
-6  -6  4
-6  -5  -4
-6  -5  -3
-6  -5  -2
-6  -5  2
-6  -5  3
-6  -5  4
-6  -4  -4
-6  -4  -3
-6  -4  -2
-6  -4  2
-6  -4  3
-6  -4  4
-6  -3  -4
-6  -3  -3
-6  -3  -2
-6  -3  2
-6  -3  3
-6  -3  4
-6  -2  -4
-6  -2  -3
-6  -2  -2
-6  -2  2
-6  -2  3
-6  -2  4
-6  0  -4
-6  0  -3
-6  0  -2
-6  0  2
-6  0  3
-6  0  4
-6  1  -4
-6  1  -3
-6  1  -2
-6  1  2
-6  1  3
-6  1  4
-6  2  -4
-6  2  -3
-6  2  -2
-6  2  2
-6  2  3
-6  2  4
-6  3  -4
-6  3  -3
-6  3  -2
-6  3  2
-6  3  3
-6  3  4
-6  4  -4
-6  4  -3
-6  4  -2
-6  4  2
-6  4  3
-6  4  4
-6  6  -4
-6  6  -3
-6  6  -2
-6  6  2
-6  6  3
-6  6  4
-5  -6  -4
-5  -6  -3
-5  -6  -2
-5  -6  2
-5  -6  3
-5  -6  4
-5  -5  -4
-5  -5  -3
-5  -5  -2
-5  -5  2
-5  -5  3
-5  -5  4
-5  -4  -4
-5  -4  -3
-5  -4  -2
-5  -4  2
-5  -4  3
-5  -4  4
-5  -3  -4
-5  -3  -3
-5  -3  -2
-5  -3  2
-5  -3  3
-5  -3  4
-5  -2  -4
-5  -2  -3
-5  -2  -2
-5  -2  2
-5  -2  3
-5  -2  4
-5  0  -4
-5  0  -3
-5  0  -2
-5  0  2
-5  0  3
-5  0  4
-5  1  -4
-5  1  -3
-5  1  -2
-5  1  2
-5  1  3
-5  1  4
-5  2  -4
-5  2  -3
-5  2  -2
-5  2  2
-5  2  3
-5  2  4
-5  3  -4
-5  3  -3
-5  3  -2
-5  3  2
-5  3  3
-5  3  4
-5  4  -4
-5  4  -3
-5  4  -2
-5  4  2
-5  4  3
-5  4  4
-5  6  -4
-5  6  -3
-5  6  -2
-5  6  2
-5  6  3
-5  6  4
-4  -6  -4
-4  -6  -3
-4  -6  -2
-4  -6  2
-4  -6  3
-4  -6  4
-4  -5  -4
-4  -5  -3
-4  -5  -2
-4  -5  2
-4  -5  3
-4  -5  4
-4  -4  -4
-4  -4  -3
-4  -4  -2
-4  -4  2
-4  -4  3
-4  -4  4
-4  -3  -4
-4  -3  -3
-4  -3  -2
-4  -3  2
-4  -3  3
-4  -3  4
-4  -2  -4
-4  -2  -3
-4  -2  -2
-4  -2  2
-4  -2  3
-4  -2  4
-4  0  -4
-4  0  -3
-4  0  -2
-4  0  2
-4  0  3
-4  0  4
-4  1  -4
-4  1  -3
-4  1  -2
-4  1  2
-4  1  3
-4  1  4
-4  2  -4
-4  2  -3
-4  2  -2
-4  2  2
-4  2  3
-4  2  4
-4  3  -4
-4  3  -3
-4  3  -2
-4  3  2
-4  3  3
-4  3  4
-4  4  -4
-4  4  -3
-4  4  -2
-4  4  2
-4  4  3
-4  4  4
-4  6  -4
-4  6  -3
-4  6  -2
-4  6  2
-4  6  3
-4  6  4
-3  -6  -4
-3  -6  -3
-3  -6  -2
-3  -6  2
-3  -6  3
-3  -6  4
-3  -5  -4
-3  -5  -3
-3  -5  -2
-3  -5  2
-3  -5  3
-3  -5  4
-3  -4  -4
-3  -4  -3
-3  -4  -2
-3  -4  2
-3  -4  3
-3  -4  4
-3  -3  -4
-3  -3  -3
-3  -3  -2
-3  -3  2
-3  -3  3
-3  -3  4
-3  -2  -4
-3  -2  -3
-3  -2  -2
-3  -2  2
-3  -2  3
-3  -2  4
-3  0  -4
-3  0  -3
-3  0  -2
-3  0  2
-3  0  3
-3  0  4
-3  1  -4
-3  1  -3
-3  1  -2
-3  1  2
-3  1  3
-3  1  4
-3  2  -4
-3  2  -3
-3  2  -2
-3  2  2
-3  2  3
-3  2  4
-3  3  -4
-3  3  -3
-3  3  -2
-3  3  2
-3  3  3
-3  3  4
-3  4  -4
-3  4  -3
-3  4  -2
-3  4  2
-3  4  3
-3  4  4
-3  6  -4
-3  6  -3
-3  6  -2
-3  6  2
-3  6  3
-3  6  4
-2  -6  -4
-2  -6  -3
-2  -6  -2
-2  -6  2
-2  -6  3
-2  -6  4
-2  -5  -4
-2  -5  -3
-2  -5  -2
-2  -5  2
-2  -5  3
-2  -5  4
-2  -4  -4
-2  -4  -3
-2  -4  -2
-2  -4  2
-2  -4  3
-2  -4  4
-2  -3  -4
-2  -3  -3
-2  -3  -2
-2  -3  2
-2  -3  3
-2  -3  4
-2  -2  -4
-2  -2  -3
-2  -2  -2
-2  -2  2
-2  -2  3
-2  -2  4
-2  0  -4
-2  0  -3
-2  0  -2
-2  0  2
-2  0  3
-2  0  4
-2  1  -4
-2  1  -3
-2  1  -2
-2  1  2
-2  1  3
-2  1  4
-2  2  -4
-2  2  -3
-2  2  -2
-2  2  2
-2  2  3
-2  2  4
-2  3  -4
-2  3  -3
-2  3  -2
-2  3  2
-2  3  3
-2  3  4
-2  4  -4
-2  4  -3
-2  4  -2
-2  4  2
-2  4  3
-2  4  4
-2  6  -4
-2  6  -3
-2  6  -2
-2  6  2
-2  6  3
-2  6  4
-1  -6  -4
-1  -6  -3
-1  -6  -2
-1  -6  2
-1  -6  3
-1  -6  4
-1  -5  -4
-1  -5  -3
-1  -5  -2
-1  -5  2
-1  -5  3
-1  -5  4
-1  -4  -4
-1  -4  -3
-1  -4  -2
-1  -4  2
-1  -4  3
-1  -4  4
-1  -3  -4
-1  -3  -3
-1  -3  -2
-1  -3  2
-1  -3  3
-1  -3  4
-1  -2  -4
-1  -2  -3
-1  -2  -2
-1  -2  2
-1  -2  3
-1  -2  4
-1  0  -4
-1  0  -3
-1  0  -2
-1  0  2
-1  0  3
-1  0  4
-1  1  -4
-1  1  -3
-1  1  -2
-1  1  2
-1  1  3
-1  1  4
-1  2  -4
-1  2  -3
-1  2  -2
-1  2  2
-1  2  3
-1  2  4
-1  3  -4
-1  3  -3
-1  3  -2
-1  3  2
-1  3  3
-1  3  4
-1  4  -4
-1  4  -3
-1  4  -2
-1  4  2
-1  4  3
-1  4  4
-1  6  -4
-1  6  -3
-1  6  -2
-1  6  2
-1  6  3
-1  6  4
0  -6  -4
0  -6  -3
0  -6  -2
0  -6  2
0  -6  3
0  -6  4
0  -5  -4
0  -5  -3
0  -5  -2
0  -5  2
0  -5  3
0  -5  4
0  -4  -4
0  -4  -3
0  -4  -2
0  -4  2
0  -4  3
0  -4  4
0  -3  -4
0  -3  -3
0  -3  -2
0  -3  2
0  -3  3
0  -3  4
0  -2  -4
0  -2  -3
0  -2  -2
0  -2  2
0  -2  3
0  -2  4
0  0  -4
0  0  -3
0  0  -2
0  0  2
0  0  3
0  0  4
0  1  -4
0  1  -3
0  1  -2
0  1  2
0  1  3
0  1  4
0  2  -4
0  2  -3
0  2  -2
0  2  2
0  2  3
0  2  4
0  3  -4
0  3  -3
0  3  -2
0  3  2
0  3  3
0  3  4
0  4  -4
0  4  -3
0  4  -2
0  4  2
0  4  3
0  4  4
0  6  -4
0  6  -3
0  6  -2
0  6  2
0  6  3
0  6  4
1  -6  -4
1  -6  -3
1  -6  -2
1  -6  2
1  -6  3
1  -6  4
1  -5  -4
1  -5  -3
1  -5  -2
1  -5  2
1  -5  3
1  -5  4
1  -4  -4
1  -4  -3
1  -4  -2
1  -4  2
1  -4  3
1  -4  4
1  -3  -4
1  -3  -3
1  -3  -2
1  -3  2
1  -3  3
1  -3  4
1  -2  -4
1  -2  -3
1  -2  -2
1  -2  2
1  -2  3
1  -2  4
1  0  -4
1  0  -3
1  0  -2
1  0  2
1  0  3
1  0  4
1  1  -4
1  1  -3
1  1  -2
1  1  2
1  1  3
1  1  4
1  2  -4
1  2  -3
1  2  -2
1  2  2
1  2  3
1  2  4
1  3  -4
1  3  -3
1  3  -2
1  3  2
1  3  3
1  3  4
1  4  -4
1  4  -3
1  4  -2
1  4  2
1  4  3
1  4  4
1  6  -4
1  6  -3
1  6  -2
1  6  2
1  6  3
1  6  4
2  -6  -4
2  -6  -3
2  -6  -2
2  -6  2
2  -6  3
2  -6  4
2  -5  -4
2  -5  -3
2  -5  -2
2  -5  2
2  -5  3
2  -5  4
2  -4  -4
2  -4  -3
2  -4  -2
2  -4  2
2  -4  3
2  -4  4
2  -3  -4
2  -3  -3
2  -3  -2
2  -3  2
2  -3  3
2  -3  4
2  -2  -4
2  -2  -3
2  -2  -2
2  -2  2
2  -2  3
2  -2  4
2  0  -4
2  0  -3
2  0  -2
2  0  2
2  0  3
2  0  4
2  1  -4
2  1  -3
2  1  -2
2  1  2
2  1  3
2  1  4
2  2  -4
2  2  -3
2  2  -2
2  2  2
2  2  3
2  2  4
2  3  -4
2  3  -3
2  3  -2
2  3  2
2  3  3
2  3  4
2  4  -4
2  4  -3
2  4  -2
2  4  2
2  4  3
2  4  4
2  6  -4
2  6  -3
2  6  -2
2  6  2
2  6  3
2  6  4
3  -6  -4
3  -6  -3
3  -6  -2
3  -6  2
3  -6  3
3  -6  4
3  -5  -4
3  -5  -3
3  -5  -2
3  -5  2
3  -5  3
3  -5  4
3  -4  -4
3  -4  -3
3  -4  -2
3  -4  2
3  -4  3
3  -4  4
3  -3  -4
3  -3  -3
3  -3  -2
3  -3  2
3  -3  3
3  -3  4
3  -2  -4
3  -2  -3
3  -2  -2
3  -2  2
3  -2  3
3  -2  4
3  0  -4
3  0  -3
3  0  -2
3  0  2
3  0  3
3  0  4
3  1  -4
3  1  -3
3  1  -2
3  1  2
3  1  3
3  1  4
3  2  -4
3  2  -3
3  2  -2
3  2  2
3  2  3
3  2  4
3  3  -4
3  3  -3
3  3  -2
3  3  2
3  3  3
3  3  4
3  4  -4
3  4  -3
3  4  -2
3  4  2
3  4  3
3  4  4
3  6  -4
3  6  -3
3  6  -2
3  6  2
3  6  3
3  6  4
4  -6  -4
4  -6  -3
4  -6  -2
4  -6  2
4  -6  3
4  -6  4
4  -5  -4
4  -5  -3
4  -5  -2
4  -5  2
4  -5  3
4  -5  4
4  -4  -4
4  -4  -3
4  -4  -2
4  -4  2
4  -4  3
4  -4  4
4  -3  -4
4  -3  -3
4  -3  -2
4  -3  2
4  -3  3
4  -3  4
4  -2  -4
4  -2  -3
4  -2  -2
4  -2  2
4  -2  3
4  -2  4
4  0  -4
4  0  -3
4  0  -2
4  0  2
4  0  3
4  0  4
4  1  -4
4  1  -3
4  1  -2
4  1  2
4  1  3
4  1  4
4  2  -4
4  2  -3
4  2  -2
4  2  2
4  2  3
4  2  4
4  3  -4
4  3  -3
4  3  -2
4  3  2
4  3  3
4  3  4
4  4  -4
4  4  -3
4  4  -2
4  4  2
4  4  3
4  4  4
4  6  -4
4  6  -3
4  6  -2
4  6  2
4  6  3
4  6  4
5  -6  -4
5  -6  -3
5  -6  -2
5  -6  2
5  -6  3
5  -6  4
5  -5  -4
5  -5  -3
5  -5  -2
5  -5  2
5  -5  3
5  -5  4
5  -4  -4
5  -4  -3
5  -4  -2
5  -4  2
5  -4  3
5  -4  4
5  -3  -4
5  -3  -3
5  -3  -2
5  -3  2
5  -3  3
5  -3  4
5  -2  -4
5  -2  -3
5  -2  -2
5  -2  2
5  -2  3
5  -2  4
5  0  -4
5  0  -3
5  0  -2
5  0  2
5  0  3
5  0  4
5  1  -4
5  1  -3
5  1  -2
5  1  2
5  1  3
5  1  4
5  2  -4
5  2  -3
5  2  -2
5  2  2
5  2  3
5  2  4
5  3  -4
5  3  -3
5  3  -2
5  3  2
5  3  3
5  3  4
5  4  -4
5  4  -3
5  4  -2
5  4  2
5  4  3
5  4  4
5  6  -4
5  6  -3
5  6  -2
5  6  2
5  6  3
5  6  4
6  -6  -4
6  -6  -3
6  -6  -2
6  -6  2
6  -6  3
6  -6  4
6  -5  -4
6  -5  -3
6  -5  -2
6  -5  2
6  -5  3
6  -5  4
6  -4  -4
6  -4  -3
6  -4  -2
6  -4  2
6  -4  3
6  -4  4
6  -3  -4
6  -3  -3
6  -3  -2
6  -3  2
6  -3  3
6  -3  4
6  -2  -4
6  -2  -3
6  -2  -2
6  -2  2
6  -2  3
6  -2  4
6  0  -4
6  0  -3
6  0  -2
6  0  2
6  0  3
6  0  4
6  1  -4
6  1  -3
6  1  -2
6  1  2
6  1  3
6  1  4
6  2  -4
6  2  -3
6  2  -2
6  2  2
6  2  3
6  2  4
6  3  -4
6  3  -3
6  3  -2
6  3  2
6  3  3
6  3  4
6  4  -4
6  4  -3
6  4  -2
6  4  2
6  4  3
6  4  4
6  6  -4
6  6  -3
6  6  -2
6  6  2
6  6  3
6  6  4
```
