# Supplementary material for: Conformational Changes in Protein Loops and Helices Induced by Post-Translational Phosphorylation
Source: PLoS Comput Biol. 2006 Apr 21;2(4):e32. doi: 10.1371/journal.pcbi.0020032 (PMC1440919; doi:10.1371/journal.pcbi.0020032)
Supplement: Supplementary file 8 [file pcbi.0020032.st008.doc]

Conformational Changes in Protein Loops and Helices Induced by Post-Translational Phosphorylation

E. S. Groban, A. Narayanan, and M. P. Jacobson

**Supporting Information**

**Supplementary Table 8**: Rotamer library used for sampling pAsp. Numbers should be multiplied by 30 to obtain the torsion angle in degrees. The columns represent the sampled heavy-atom torsion angles, other than the rotation about the O-P bond in the phosphate group, which is sampled uniformly. χ_1_ represents rotation about the C_α_-C_β_ bond, χ_2_ is the rotation about C_β_-C_γ_, and χ_3_ is rotation about the C_γ_-O bond.

```
-5  -5  -5
-5  -5  -1
-5  -5  0
-5  -5  1
-5  -5  2
-5  -5  5
-5  -5  6
-5  -4  -5
-5  -4  -1
-5  -4  0
-5  -4  1
-5  -4  2
-5  -4  5
-5  -4  6
-5  -3  -5
-5  -3  -1
-5  -3  0
-5  -3  1
-5  -3  2
-5  -3  5
-5  -3  6
-5  -2  -5
-5  -2  -1
-5  -2  0
-5  -2  1
-5  -2  2
-5  -2  5
-5  -2  6
-5  -1  -5
-5  -1  -1
-5  -1  0
-5  -1  1
-5  -1  2
-5  -1  5
-5  -1  6
-5  0  -5
-5  0  -1
-5  0  0
-5  0  1
-5  0  2
-5  0  5
-5  0  6
-5  1  -5
-5  1  -1
-5  1  0
-5  1  1
-5  1  2
-5  1  5
-5  1  6
-5  2  -5
-5  2  -1
-5  2  0
-5  2  1
-5  2  2
-5  2  5
-5  2  6
-5  3  -5
-5  3  -1
-5  3  0
-5  3  1
-5  3  2
-5  3  5
-5  3  6
-5  4  -5
-5  4  -1
-5  4  0
-5  4  1
-5  4  2
-5  4  5
-5  4  6
-5  5  -5
-5  5  -1
-5  5  0
-5  5  1
-5  5  2
-5  5  5
-5  5  6
-5  6  -5
-5  6  -1
-5  6  0
-5  6  1
-5  6  2
-5  6  5
-5  6  6
-4  -5  -5
-4  -5  -1
-4  -5  0
-4  -5  1
-4  -5  2
-4  -5  5
-4  -5  6
-4  -4  -5
-4  -4  -1
-4  -4  0
-4  -4  1
-4  -4  2
-4  -4  5
-4  -4  6
-4  -3  -5
-4  -3  -1
-4  -3  0
-4  -3  1
-4  -3  2
-4  -3  5
-4  -3  6
-4  -2  -5
-4  -2  -1
-4  -2  0
-4  -2  1
-4  -2  2
-4  -2  5
-4  -2  6
-4  -1  -5
-4  -1  -1
-4  -1  0
-4  -1  1
-4  -1  2
-4  -1  5
-4  -1  6
-4  0  -5
-4  0  -1
-4  0  0
-4  0  1
-4  0  2
-4  0  5
-4  0  6
-4  1  -5
-4  1  -1
-4  1  0
-4  1  1
-4  1  2
-4  1  5
-4  1  6
-4  2  -5
-4  2  -1
-4  2  0
-4  2  1
-4  2  2
-4  2  5
-4  2  6
-4  3  -5
-4  3  -1
-4  3  0
-4  3  1
-4  3  2
-4  3  5
-4  3  6
-4  4  -5
-4  4  -1
-4  4  0
-4  4  1
-4  4  2
-4  4  5
-4  4  6
-4  5  -5
-4  5  -1
-4  5  0
-4  5  1
-4  5  2
-4  5  5
-4  5  6
-4  6  -5
-4  6  -1
-4  6  0
-4  6  1
-4  6  2
-4  6  5
-4  6  6
-3  -5  -5
-3  -5  -1
-3  -5  0
-3  -5  1
-3  -5  2
-3  -5  5
-3  -5  6
-3  -4  -5
-3  -4  -1
-3  -4  0
-3  -4  1
-3  -4  2
-3  -4  5
-3  -4  6
-3  -3  -5
-3  -3  -1
-3  -3  0
-3  -3  1
-3  -3  2
-3  -3  5
-3  -3  6
-3  -2  -5
-3  -2  -1
-3  -2  0
-3  -2  1
-3  -2  2
-3  -2  5
-3  -2  6
-3  -1  -5
-3  -1  -1
-3  -1  0
-3  -1  1
-3  -1  2
-3  -1  5
-3  -1  6
-3  0  -5
-3  0  -1
-3  0  0
-3  0  1
-3  0  2
-3  0  5
-3  0  6
-3  1  -5
-3  1  -1
-3  1  0
-3  1  1
-3  1  2
-3  1  5
-3  1  6
-3  2  -5
-3  2  -1
-3  2  0
-3  2  1
-3  2  2
-3  2  5
-3  2  6
-3  3  -5
-3  3  -1
-3  3  0
-3  3  1
-3  3  2
-3  3  5
-3  3  6
-3  4  -5
-3  4  -1
-3  4  0
-3  4  1
-3  4  2
-3  4  5
-3  4  6
-3  5  -5
-3  5  -1
-3  5  0
-3  5  1
-3  5  2
-3  5  5
-3  5  6
-3  6  -5
-3  6  -1
-3  6  0
-3  6  1
-3  6  2
-3  6  5
-3  6  6
-2  -5  -5
-2  -5  -1
-2  -5  0
-2  -5  1
-2  -5  2
-2  -5  5
-2  -5  6
-2  -4  -5
-2  -4  -1
-2  -4  0
-2  -4  1
-2  -4  2
-2  -4  5
-2  -4  6
-2  -3  -5
-2  -3  -1
-2  -3  0
-2  -3  1
-2  -3  2
-2  -3  5
-2  -3  6
-2  -2  -5
-2  -2  -1
-2  -2  0
-2  -2  1
-2  -2  2
-2  -2  5
-2  -2  6
-2  -1  -5
-2  -1  -1
-2  -1  0
-2  -1  1
-2  -1  2
-2  -1  5
-2  -1  6
-2  0  -5
-2  0  -1
-2  0  0
-2  0  1
-2  0  2
-2  0  5
-2  0  6
-2  1  -5
-2  1  -1
-2  1  0
-2  1  1
-2  1  2
-2  1  5
-2  1  6
-2  2  -5
-2  2  -1
-2  2  0
-2  2  1
-2  2  2
-2  2  5
-2  2  6
-2  3  -5
-2  3  -1
-2  3  0
-2  3  1
-2  3  2
-2  3  5
-2  3  6
-2  4  -5
-2  4  -1
-2  4  0
-2  4  1
-2  4  2
-2  4  5
-2  4  6
-2  5  -5
-2  5  -1
-2  5  0
-2  5  1
-2  5  2
-2  5  5
-2  5  6
-2  6  -5
-2  6  -1
-2  6  0
-2  6  1
-2  6  2
-2  6  5
-2  6  6
-1  -5  -5
-1  -5  -1
-1  -5  0
-1  -5  1
-1  -5  2
-1  -5  5
-1  -5  6
-1  -4  -5
-1  -4  -1
-1  -4  0
-1  -4  1
-1  -4  2
-1  -4  5
-1  -4  6
-1  -3  -5
-1  -3  -1
-1  -3  0
-1  -3  1
-1  -3  2
-1  -3  5
-1  -3  6
-1  -2  -5
-1  -2  -1
-1  -2  0
-1  -2  1
-1  -2  2
-1  -2  5
-1  -2  6
-1  -1  -5
-1  -1  -1
-1  -1  0
-1  -1  1
-1  -1  2
-1  -1  5
-1  -1  6
-1  0  -5
-1  0  -1
-1  0  0
-1  0  1
-1  0  2
-1  0  5
-1  0  6
-1  1  -5
-1  1  -1
-1  1  0
-1  1  1
-1  1  2
-1  1  5
-1  1  6
-1  2  -5
-1  2  -1
-1  2  0
-1  2  1
-1  2  2
-1  2  5
-1  2  6
-1  3  -5
-1  3  -1
-1  3  0
-1  3  1
-1  3  2
-1  3  5
-1  3  6
-1  4  -5
-1  4  -1
-1  4  0
-1  4  1
-1  4  2
-1  4  5
-1  4  6
-1  5  -5
-1  5  -1
-1  5  0
-1  5  1
-1  5  2
-1  5  5
-1  5  6
-1  6  -5
-1  6  -1
-1  6  0
-1  6  1
-1  6  2
-1  6  5
-1  6  6
0  -5  -5
0  -5  -1
0  -5  0
0  -5  1
0  -5  2
0  -5  5
0  -5  6
0  -4  -5
0  -4  -1
0  -4  0
0  -4  1
0  -4  2
0  -4  5
0  -4  6
0  -3  -5
0  -3  -1
0  -3  0
0  -3  1
0  -3  2
0  -3  5
0  -3  6
0  -2  -5
0  -2  -1
0  -2  0
0  -2  1
0  -2  2
0  -2  5
0  -2  6
0  -1  -5
0  -1  -1
0  -1  0
0  -1  1
0  -1  2
0  -1  5
0  -1  6
0  0  -5
0  0  -1
0  0  0
0  0  1
0  0  2
0  0  5
0  0  6
0  1  -5
0  1  -1
0  1  0
0  1  1
0  1  2
0  1  5
0  1  6
0  2  -5
0  2  -1
0  2  0
0  2  1
0  2  2
0  2  5
0  2  6
0  3  -5
0  3  -1
0  3  0
0  3  1
0  3  2
0  3  5
0  3  6
0  4  -5
0  4  -1
0  4  0
0  4  1
0  4  2
0  4  5
0  4  6
0  5  -5
0  5  -1
0  5  0
0  5  1
0  5  2
0  5  5
0  5  6
0  6  -5
0  6  -1
0  6  0
0  6  1
0  6  2
0  6  5
0  6  6
1  -5  -5
1  -5  -1
1  -5  0
1  -5  1
1  -5  2
1  -5  5
1  -5  6
1  -4  -5
1  -4  -1
1  -4  0
1  -4  1
1  -4  2
1  -4  5
1  -4  6
1  -3  -5
1  -3  -1
1  -3  0
1  -3  1
1  -3  2
1  -3  5
1  -3  6
1  -2  -5
1  -2  -1
1  -2  0
1  -2  1
1  -2  2
1  -2  5
1  -2  6
1  -1  -5
1  -1  -1
1  -1  0
1  -1  1
1  -1  2
1  -1  5
1  -1  6
1  0  -5
1  0  -1
1  0  0
1  0  1
1  0  2
1  0  5
1  0  6
1  1  -5
1  1  -1
1  1  0
1  1  1
1  1  2
1  1  5
1  1  6
1  2  -5
1  2  -1
1  2  0
1  2  1
1  2  2
1  2  5
1  2  6
1  3  -5
1  3  -1
1  3  0
1  3  1
1  3  2
1  3  5
1  3  6
1  4  -5
1  4  -1
1  4  0
1  4  1
1  4  2
1  4  5
1  4  6
1  5  -5
1  5  -1
1  5  0
1  5  1
1  5  2
1  5  5
1  5  6
1  6  -5
1  6  -1
1  6  0
1  6  1
1  6  2
1  6  5
1  6  6
2  -5  -5
2  -5  -1
2  -5  0
2  -5  1
2  -5  2
2  -5  5
2  -5  6
2  -4  -5
2  -4  -1
2  -4  0
2  -4  1
2  -4  2
2  -4  5
2  -4  6
2  -3  -5
2  -3  -1
2  -3  0
2  -3  1
2  -3  2
2  -3  5
2  -3  6
2  -2  -5
2  -2  -1
2  -2  0
2  -2  1
2  -2  2
2  -2  5
2  -2  6
2  -1  -5
2  -1  -1
2  -1  0
2  -1  1
2  -1  2
2  -1  5
2  -1  6
2  0  -5
2  0  -1
2  0  0
2  0  1
2  0  2
2  0  5
2  0  6
2  1  -5
2  1  -1
2  1  0
2  1  1
2  1  2
2  1  5
2  1  6
2  2  -5
2  2  -1
2  2  0
2  2  1
2  2  2
2  2  5
2  2  6
2  3  -5
2  3  -1
2  3  0
2  3  1
2  3  2
2  3  5
2  3  6
2  4  -5
2  4  -1
2  4  0
2  4  1
2  4  2
2  4  5
2  4  6
2  5  -5
2  5  -1
2  5  0
2  5  1
2  5  2
2  5  5
2  5  6
2  6  -5
2  6  -1
2  6  0
2  6  1
2  6  2
2  6  5
2  6  6
3  -5  -5
3  -5  -1
3  -5  0
3  -5  1
3  -5  2
3  -5  5
3  -5  6
3  -4  -5
3  -4  -1
3  -4  0
3  -4  1
3  -4  2
3  -4  5
3  -4  6
3  -3  -5
3  -3  -1
3  -3  0
3  -3  1
3  -3  2
3  -3  5
3  -3  6
3  -2  -5
3  -2  -1
3  -2  0
3  -2  1
3  -2  2
3  -2  5
3  -2  6
3  -1  -5
3  -1  -1
3  -1  0
3  -1  1
3  -1  2
3  -1  5
3  -1  6
3  0  -5
3  0  -1
3  0  0
3  0  1
3  0  2
3  0  5
3  0  6
3  1  -5
3  1  -1
3  1  0
3  1  1
3  1  2
3  1  5
3  1  6
3  2  -5
3  2  -1
3  2  0
3  2  1
3  2  2
3  2  5
3  2  6
3  3  -5
3  3  -1
3  3  0
3  3  1
3  3  2
3  3  5
3  3  6
3  4  -5
3  4  -1
3  4  0
3  4  1
3  4  2
3  4  5
3  4  6
3  5  -5
3  5  -1
3  5  0
3  5  1
3  5  2
3  5  5
3  5  6
3  6  -5
3  6  -1
3  6  0
3  6  1
3  6  2
3  6  5
3  6  6
4  -5  -5
4  -5  -1
4  -5  0
4  -5  1
4  -5  2
4  -5  5
4  -5  6
4  -4  -5
4  -4  -1
4  -4  0
4  -4  1
4  -4  2
4  -4  5
4  -4  6
4  -3  -5
4  -3  -1
4  -3  0
4  -3  1
4  -3  2
4  -3  5
4  -3  6
4  -2  -5
4  -2  -1
4  -2  0
4  -2  1
4  -2  2
4  -2  5
4  -2  6
4  -1  -5
4  -1  -1
4  -1  0
4  -1  1
4  -1  2
4  -1  5
4  -1  6
4  0  -5
4  0  -1
4  0  0
4  0  1
4  0  2
4  0  5
4  0  6
4  1  -5
4  1  -1
4  1  0
4  1  1
4  1  2
4  1  5
4  1  6
4  2  -5
4  2  -1
4  2  0
4  2  1
4  2  2
4  2  5
4  2  6
4  3  -5
4  3  -1
4  3  0
4  3  1
4  3  2
4  3  5
4  3  6
4  4  -5
4  4  -1
4  4  0
4  4  1
4  4  2
4  4  5
4  4  6
4  5  -5
4  5  -1
4  5  0
4  5  1
4  5  2
4  5  5
4  5  6
4  6  -5
4  6  -1
4  6  0
4  6  1
4  6  2
4  6  5
4  6  6
5  -5  -5
5  -5  -1
5  -5  0
5  -5  1
5  -5  2
5  -5  5
5  -5  6
5  -4  -5
5  -4  -1
5  -4  0
5  -4  1
5  -4  2
5  -4  5
5  -4  6
5  -3  -5
5  -3  -1
5  -3  0
5  -3  1
5  -3  2
5  -3  5
5  -3  6
5  -2  -5
5  -2  -1
5  -2  0
5  -2  1
5  -2  2
5  -2  5
5  -2  6
5  -1  -5
5  -1  -1
5  -1  0
5  -1  1
5  -1  2
5  -1  5
5  -1  6
5  0  -5
5  0  -1
5  0  0
5  0  1
5  0  2
5  0  5
5  0  6
5  1  -5
5  1  -1
5  1  0
5  1  1
5  1  2
5  1  5
5  1  6
5  2  -5
5  2  -1
5  2  0
5  2  1
5  2  2
5  2  5
5  2  6
5  3  -5
5  3  -1
5  3  0
5  3  1
5  3  2
5  3  5
5  3  6
5  4  -5
5  4  -1
5  4  0
5  4  1
5  4  2
5  4  5
5  4  6
5  5  -5
5  5  -1
5  5  0
5  5  1
5  5  2
5  5  5
5  5  6
5  6  -5
5  6  -1
5  6  0
5  6  1
5  6  2
5  6  5
5  6  6
6  -5  -5
6  -5  -1
6  -5  0
6  -5  1
6  -5  2
6  -5  5
6  -5  6
6  -4  -5
6  -4  -1
6  -4  0
6  -4  1
6  -4  2
6  -4  5
6  -4  6
6  -3  -5
6  -3  -1
6  -3  0
6  -3  1
6  -3  2
6  -3  5
6  -3  6
6  -2  -5
6  -2  -1
6  -2  0
6  -2  1
6  -2  2
6  -2  5
6  -2  6
6  -1  -5
6  -1  -1
6  -1  0
6  -1  1
6  -1  2
6  -1  5
6  -1  6
6  0  -5
6  0  -1
6  0  0
6  0  1
6  0  2
6  0  5
6  0  6
6  1  -5
6  1  -1
6  1  0
6  1  1
6  1  2
6  1  5
6  1  6
6  2  -5
6  2  -1
6  2  0
6  2  1
6  2  2
6  2  5
6  2  6
6  3  -5
6  3  -1
6  3  0
6  3  1
6  3  2
6  3  5
6  3  6
6  4  -5
6  4  -1
6  4  0
6  4  1
6  4  2
6  4  5
6  4  6
6  5  -5
6  5  -1
6  5  0
6  5  1
6  5  2
6  5  5
6  5  6
6  6  -5
6  6  -1
6  6  0
6  6  1
6  6  2
6  6  5
```

6 6 6
